# Regulating quasi-legal markets: Evidence from pain management clinic laws

**DOI:** 10.1016/j.jpubeco.2025.105515

**Published:** 2025-11-18

**Authors:** Yuji Mizushima, David Powell, Rahi Abouk, Cheryl Damberg

**Affiliations:** a RAND, United States; b University of Pennsylvania, United States; c William Paterson University, United States

**Keywords:** I11, I12, I18, Opioid crisis, Prescribing behavior, Opioid dispensing, Pain management clinics

## Abstract

The opioid crisis has often been fueled by its simultaneous interaction with both medical and illicit markets, including “pill mills” that distribute legal substances in inappropriate and quasi-legal ways. Pain management clinic laws (PMCLs) aim to address this property by enforcing stricter regulatory licensing requirements and regulatory oversight on opioid prescribing establishments. Using a difference-in-differences framework and Medicare claims data, we find that PMCLs reduce problematic opioid prescribing and doctor shopping. Drawing on transaction-level information on opioid shipments, we estimate that PMCLs lead to strikingly large reductions in the volume of opioids dispensed directly by practitioners to patients. Studying mortality data, we estimate reductions in overdose death rates involving prescription opioids, with little evidence of substitution to illicit opioid markets. As PMCLs have not been adopted in most states, our results suggest they warrant greater attention from policymakers, even amid the declining role of prescription opioids in the annual death toll of the opioid crisis.

## 1. Introduction

The opioid crisis represents the worst drug crisis in the history of the United States. A driving force of the opioid crisis has been the emergence of high-potency opioids at the intersection of legal and illicit markets. Opioids are accessible both medically and illicitly, and the distribution of prescription opioids has sometimes blurred the distinction between the two (Bradford & Lozano-Rojas, 2023). “Pill mills” operate in this quasi-legal status by prescribing legal drugs but doing so at inappropriate levels and without due consideration for patient pain management needs. Because “medically appropriate” prescribing includes subjectivity and deference to the prescribers, pill mills can be difficult to designate, legislate, and police (Twillman, 2012). However, they have also been thought to be important factors fueling the opioid crisis (Carroll et al., 2018; Clark & Schumacher, 2017; Temple, 2015), and they remain a major problem.^1^

Their importance continues even as the opioid crisis itself transitions to heroin and illicitly-manufactured fentanyl given that it is common to use or misuse prescription opioids prior to initiating illicit opioids (Compton et al., 2016; Powell & Jacobson, 2025). Not all pain management clinics are pill mills. However, some states have targeted pain management clinics through legislation to identify, regulate, and deter pill mills from operating. These states have implemented pain management clinic laws (PMCLs) which subject certain types of medical practices to stricter regulations (Maclean et al., 2021).

Unlike many policies governing prescription opioids, the intention of PMCLs is to address inappropriate prescription opioid distribution in quasi-legal settings. As an investigative report documented in Temple (2015) stated, “[T]he problem for law enforcement is that what these clinics are doing is perfectly legal” (p. 97). The regulations imposed by PMCLs provide an opportunity to understand a policy option which regulates a component of the illicit market, as pain management clinics have been important sources of the illicit opioid supply through the diversion of prescription opioids from medically-intended purposes (Cicero & Inciardi, 2005; Rigg et al., 2010; Surratt et al., 2014; Temple, 2015). The proximity of pill mills to the illicit market suggests that PMCLs may succeed where other supply-side interventions have failed. Alternatively, pill mills may serve a population which is closer to the margin of transitioning to illicit markets, implying scope for the unintended consequences often associated with supply-side interventions (Kim, 2021; Mallatt, 2022; Meinhofer, 2018).

Despite the reported importance of pill mills throughout the history of the opioid crisis, the literature on PMCLs is relatively limited with most studies focused on laws passed in Florida in 2010–2011 (Kennedy-Hendricks et al., 2016; Rutkow et al., 2015; Surratt et al., 2014) or in Texas in 2010 (Lyapustina et al., 2016). There are few multi-state evaluations and they typically study broad measures of total state opioid supply or overdose rates (Brighthaupt et al., 2019; Cerdá et al., 2021; Frizzell et al., 2020; Kaestner & Ziedan, 2023; Liang & Shi, 2020; Popovici et al., 2018).^2^ There are over four times as many studies on prescription drug monitoring programs (PDMPs) (Schuler et al., 2020). There is broad interest in understanding the role of regulatory oversight and enforcement in addressing opioid overprescribing, with recent papers finding that DEA enforcement actions targeting specific practices or pharmacies impact aggregate measures of opioid dispensing (Donahoe, 2022; Soliman, 2025).

In this paper, we evaluate a wide-ranging and informative set of dispensing- and prescribing-related outcomes, including metrics providing evidence about heavy opioid use and doctor shopping. We use the census of the Medicare Part D claims data to construct rich prescribing measures. In addition to standard measures such as number of opioid prescriptions and days supplied, we examine high levels of opioid prescribing over the course of several months, the distribution of prescription lengths, and daily dosage levels of prescriptions. These measures help provide a comprehensive assessment about how opioid prescribing is impacted.

While specific to the Social Security Disability Insurance (SSDI) and age 65+ populations, the literature on opioid-targeting policies has found Medicare data especially useful (Bradford et al., 2018; Buchmueller & Carey, 2018; Moyo et al., 2017) and there is no research on the effect of PMCLs among government-sponsored healthcare coverage beneficiaries such as Medicare enrollees. The SSDI population, in particular, has high rates of opioid use (Cutler et al., 2017; Maestas et al., 2021; Morden et al., 2014) and is at high risk of overdose (Peters et al., 2018; Song, 2017). Opioid prescribing to the Medicare population is also important due to evidence that access to opioids through Medicare has substantial spillovers to the rest of the population through high levels of diversion, suggesting that prescribing behavior for this population has meaningful, troubling externalities (Knowles, 2022; Powell et al., 2020).

One motivation for studying policy levers which may impact prescribing behavior is due to the ability of policy to more directly address inappropriate opioid access through medical channels relative to illicit channels. Even as illicit opioids drive the opioid crisis in recent years, there is still a role for reducing initiation and dependence through prescribing behavior (Powell & Jacobson, 2025). We will examine prescriptions to opioid-naïve patients for exactly this reason. Moreover, there are concerns that many opioid-restricting policies are too blunt, reducing access to a key pain management tool for suffering patients (Chua et al., 2019). We construct and study measures to help assess whether PMCLs broadly reduce opioid prescriptions or appear to target more problematic prescribing behavior.

We complement this analysis by studying transaction-level data on opioid shipments to test how PMCLs affect the types of facilities dispensing opioids, focusing on practitioners directly dispensing opioids, chain pharmacies, and local pharmacies. A defining characteristic of pill mills is that they have a strong profit motive to prescribe opioids. Opioids must be both prescribed and dispensed, and pill mills typically dispense opioids directly or coordinate with a local pharmacy (in return for kickbacks) to fill inappropriate opioid prescriptions. Studying these outcomes helps us quantify how pill mills operate and how PMCLs impact opioid access. A fundamental goal of PMCLs is to eliminate or reduce the profit incentive associated with prescribing opioids, and a contribution of this paper is to test whether PMCLs appear to accomplish this goal. We also study data on establishments to test whether PMCLs lead to pain clinic closures.

Prescribing behavior and opioid shipments provide important evidence about the dissemination of opioids through legal and quasi-legal channels. However, it cannot capture transitions into illicit markets, which have historically been difficult to measure, study, and regulate. Overdose deaths potentially provide a window into these transitions, and we will study opioid-involved overdose deaths as well as more specific categories such as deaths involving prescription opioids and deaths involving heroin or synthetic opioids, which typically (but imperfectly) proxy for illicit opioids.

We use state-level PMCL adoption during the 2010–2018 time period in a difference-in-differences framework to understand the impacts of PMCL policies on our outcomes of interest. We find that PMCLs are especially effective in deterring overprescribing and doctor shopping. We estimate reductions in overall opioid prescribing metrics, and even larger proportional reductions in the share of beneficiaries receiving 121+ days of opioids in a quarter, and 211+ days in a half-year, our measures of high prescribing over longer periods of time.

As measures of doctor shopping, we study overlapping claims, frequency of new patient codes, and the number of opioid prescribers per beneficiary within a quarter. We observe substantial reductions in these measures when PMCLs are adopted. At the prescription level, we estimate disproportionately large reductions in lengthy prescriptions (22+ days) and high dosage prescriptions. In general, we estimate much stronger effects across all metrics for the SSDI population and for the opioid non-naïve population.

Our analysis of opioid shipments finds strikingly large reductions in opioids shipped to practitioners, strongly suggesting that PMCLs sever integrated prescribing and dispensing practices. Likewise, we estimate large reductions to shipments to local pharmacies with much less evidence of changes in opioid shipments to chain pharmacies. This latter set of results is consistent with qualitative reports that pill mills establish relationships with local pharmacies and avoid chain pharmacies. We find some but limited evidence that PMCLs reduce inappropriate prescribing through reductions in the number of pain management clinics, consistent with an important role for changes in the behaviors of existing pain management clinics.

Our mortality analysis suggests that PMCLs reduce prescription opioid-related overdose death rates, and we do not observe evidence of a shift to illicit markets. This result contrasts with recent work which finds increases in deaths attributable to illicit opioids after implementation of PMCLs. We explore the reasons behind this inconsistency with the literature and emphasize the need for analyses of opioid policies to consider the geography of policy adoption.

Policy solutions with meaningful effects in response to the opioid crisis have been difficult to identify (Rao et al., 2021). Our work suggests that PMCLs, which have still yet to be adopted in a majority of states, and similar enforcement activities which target suspicious prescribing sources (Donahoe, 2022; Soliman, 2025) merit substantially more attention from policymakers even in the current illicit opioid landscape. The results imply gains to specific types of supply-side interventions which more directly target inappropriate prescribing behavior at an establishment level.

The next section provides additional background. In Section 3, we introduce the data and empirical strategy. Section 4 reports the main results. We present the mortality results in Section 5 and discuss their relationship with previous findings in the literature. Section 6 concludes.

## 2. Background

### 2.1. Pill mills and the opioid crisis

Pill mills provide inappropriate access to opioids, diverting them to individuals intent on misuse and, through extensive secondhand diversion, supplying illicit markets across the country (Cicero et al., 2011; Rigg et al., 2010; Surratt et al., 2014; Temple, 2015). They prescribe opioids without adequate inspection of patient health and need for opioids, enabled by fractured regulatory oversight over the quality of care provided by pill mills.

The first wave of the opioid crisis was predominantly driven by access to prescription opioids in both medical and illicit settings. Pill mills are often faulted for their role in this era of the opioid crisis. While less common, pill mills continue to supply prescription opioids in a way that promotes misuse and feeds the illicit market (Department of Justice, 2019a, 2019b, 2020a, 2020b, 2021, 2023, 2025a, 2025b, 2025c). Given the nature of pill mills, their operations often remain hidden and require extensive investigative efforts to uncover and understand. While there are qualitative accounts of specific pill mills and how they operate, there is little empirical work which confirms this behavior on a broader scale. This study relies on journalistic and ethnographic descriptions of pill mills to construct useful measures which should be impacted by PMCLs if those accounts generalize enough to pill mills more broadly. A contribution of this paper then is testing some of the predictions that follow from qualitative research in this field.

Even prior to PMCLs, pill mills typically tried to avoid detection and would operate to avoid raising the suspicions of authorities. Temple (2015) details how one large pill mill would provide a month’s supply of pills and then “would schedule appointments no closer than twenty-eight days apart” (p. 63). This was a common practice:

“Most pain management clinics limited their patients to one visit a month. Some participants mentioned that they consistently ran out of pills before the month was up or needed to get more pills to sell to pay their bills. A common way to circumvent this waiting period was to visit more than one pain doctor, usually ones with on-site pharmacies. Visiting several pain management clinics for the purpose of obtaining multiple prescriptions was a practice that all the participants engaged in at some point in time.”

(Rigg et al., 2010)

The importance of on-site pharmacies is a common theme in accounts about pill mills. Pill mills prescribe high quantities of opioids, but they do not make most of their money by prescribing. Instead, they have to dispense the opioids themselves or use a peer pharmacy which would pay them for the business:

“Many of the pill mills also dispense narcotics on site and many patients are referred to a specific pharmacy recommended by the physician or office personnel in an attempt to mitigate contact with law enforcement. In fact, many of the physicians collude with the pharmacies by either co-owning the pharmacy or receiving kickbacks from the pharmacist.”

(Ramirez, 2011)

This attached a direct monetary incentive to the *prescribing* of opioids. Pill mills without on-site pharmacies appear to favor local pharmacies (Department of Justice, 2025b). As discussed in Rigg et al. (2010): “Prescriptions were usually filled at smaller family owned pharmacies…Large chain pharmacies were avoided.”

These behaviors provide testable predictions for how a shock to regulatory enforcement capabilities should influence the actions of prescribers and dispensers, and we use these qualitative analyses to inform the construction of our outcomes.

### 2.2. Pain management clinic laws

PMCLs represent a regulatory response to the threats posed by pill mills and permit extensive regulation of enterprises that prescribe large volumes of narcotics for pain management. While PMCLs have multiple dimensions, the common characteristic is an increase in regulatory authority over pain management clinics with the goal of reducing the economic incentives to prescribe opioids. This is accomplished primarily in two ways: (1) raising *qualifications* for clinic ownership and practice, and (2) enhancing *oversight* of clinic operations through mandatory registration, facility inspections, required clinical procedures such as physical examinations and drug testing, patient recordkeeping requirements, and restrictions on on-site dispensing.

Table 1 provides enactment dates of the PMCLs while highlighting specific dimensions of the policies. We include a more detailed set of dimensions in Table A1. While listed as a PMCL state, we exclude Arizona in our analyses. The Arizona law went into effect in 2018, but the penalty and licensure rules were not enacted until 2019 (after our sample period) and the policy had no useful regulatory oversight functions (see Table A1 for details). This leaves us with nine states adopting PMCLs during our sample period. In this section, we discuss common features of these nine PMCLs and those adopted prior to the sample period in Louisiana and Texas.

First, all PMCLs regulate establishments by increasing the *qualifications* required to both own and practice in pain management clinics. For example, eight out of 11 states with PMCLs require pain clinics to be owned by a licensed physician, eight prohibit ownership by individuals with a history of DEA license denial, seven bar owners with felony convictions, and four require owners to hold specialized credentials such as board certification in pain management. For prescribers, nine states prohibit prescribing by physicians with prior DEA license denials. PMCLs thus attempt to improve the clinical quality of decision-making by changing the underlying composition of providers who are permitted to treat pain. This may decrease the prevalence of inappropriate prescribing that would otherwise go undetected under conventional PDMP or prescribing limit regimes, which monitor prescribing volumes but not the clinical quality of decision-making.

A second key component of PMCLs is an increase in *oversight* over pain clinic practices and operations. This is achieved by requiring pain clinics to register with state medical boards, mandating regular registration renewals, and requiring a more detailed paper trail to facilitate inspections and investigations. Nine of the 11 states enable regular facility inspections, ten require prescribers to conduct a physical examination of patients prior to initiating chronic pain treatment and require clinics to keep all patient records, and seven mandate drug testing of patients prior to the prescribing of controlled substances. Requiring physicians to conduct pre-treatment evaluations creates a paper trail that promotes provider accountability and helps safeguard against the prescribing of opioids without first assessing clinical appropriateness, including potential contraindications or undisclosed indications of prior opioid use by patients. As part of broader operational oversight, five states further regulate the dispensing of opioids within pain clinics by restricting dispensing privileges to qualified providers (e.g., licensed physicians) and the quantity dispensed (e.g., 72-hour supply limits).

To ensure compliance with these regulations, states enforce PMCL provisions through formal disciplinary measures and criminal penalties. While there is heterogeneity across the different policies, this is the norm in analyses studying opioid-related and substance use policies given the diverse characteristics of state PDMPs (e.g., which substances are included in the PMDP, frequency of data reporting, etc.), state cannabis laws (see Pacula et al. (2015)), naloxone policies (e.g., standing orders, protocol orders, and prescriptive authority policies), and so on. The common thread with PMCLs is the improvement of regulatory oversight by the state. We treat PMCLs as a regulatory shock to oversight of opioid-prescribing establishments.

### 2.3. Conceptual framework

PMCLs may impact opioid access through two primary mechanisms. First, by imposing stricter licensing and credentialing requirements, PMCLs can raise the average quality of providers practicing in pain clinics. This screening function helps reduce adverse selection on the supply-side of the opioid market by excluding providers with histories of inappropriate prescribing, dispensing, or limited expertise in pain management. Second, greater oversight over the quality of pain clinic practices through regular site inspections, mandatory pre-treatment patient drug testing, and restrictions on dispensing may deter careless or profit-driven prescribing practices. This mechanism operates by addressing supply-side moral hazard in the opioid prescription market.

Consequently, changes in opioid prescribing may result from (1) changes in prescribing practices among a fixed set of pain management clinics or (2) through changes in the composition of pain management clinics through systematic closures and entry. In response to PMCLs, we expect to observe declines in opioid prescribing, driven disproportionately by reductions in metrics related to over-prescribing. We also expect to observe changes in opioid supply among establishments that might have direct or indirect ties to pill mills.

### 2.4. Pain management among the Medicare population

Much of our analysis studies the Medicare population. Medicare beneficiaries have a higher pain prevalence rate than the general population. The 2018 Medicare Current Beneficiary Survey (MCBS) reports that 78 percent of all Medicare enrollees experience chronic pain (Medicare Current Beneficiary Survey, 2020) compared to only 20 percent who struggle with chronic pain in the overall population (Yong et al., 2022). The higher prevalence of chronic pain in Medicare beneficiaries is associated with an increased prevalence of opioid use disorder (OUD) compared to the overall population. The opioid mortality rate for the Medicare beneficiaries under age-65 is nearly six times higher than that for the overall population (Kuo et al., 2019). The heightened prevalence of opioid use and OUD among the Medicare population highlights the need for paying close attention to how opioid-related policies impact this particular group. However, research on PMCLs for this group is scarce.

### 2.5. Literature on pain management clinic laws

Mallatt (2020) finds an 11 percent decline in the number of pain management clinics after PMCL adoption. Using establishment-level data including information on sales revenue, Burton and Churchill (2025) estimate that PMCLs increase pharmacy closures by approximately 4 percent and decrease pharmacy sales (measured in dollars) by 5 percent. Kaestner and Ziedan (2023) estimate that PMCLs lead to a 14–28 percent decline in aggregate state opioid supply (but no statistically significant change in mortality).^3^ State-specific analyses have suggested large reductions in opioid shipments due to PMCL adoption (Lyapustina et al., 2016; Rutkow et al., 2015).

The most similar investigation to the present study is Cerdá et al. (2021) which evaluates the effect of PMCLs on opioid overdose deaths and heavy prescription opioid use using IQVIA data from 2010 to 2018 and estimating hierarchical Bayesian spatiotemporal models. Their findings suggest that PMCLs reduced heavy prescription opioid use and overall and natural/semisynthetic opioid overdose deaths. They also find that PMCLs increased synthetic opioid and heroin deaths, implying shifts to illicit opioid markets.

Our paper makes the following notable contributions to this literature. First, this study examines a comprehensive set of opioid prescribing measures relative to the literature, allowing us to accurately characterize changes in prescribing behavior that would otherwise be difficult to capture with state opioid supply in the ARCOS data. Second, our study is the first to examine how PMCLs affect measures of doctor shopping. Anecdotal evidence (see Section 2.1) suggests that pill mills promote doctor shopping by capping the frequency of patient visits. Conceptually, however, this relationship is unclear and formal analyses are needed to complement the anecdotal record. Our study paints a broader picture of the various intended and unintended prescribing and health consequences of PMCLs, heterogeneity across various sub-populations, and the mechanisms underlying them, compared to the prior literature. Third, we are the first to study how PMCLs impact the integration of prescribing and dispensing by studying opioid shipments by establishment type. Pill mills depend on profiting directly from the prescribing of opioids, yet outcomes related to this core feature have never been studied.

Fourth, much of the cited literature does not address concerns related to treatment heterogeneity given staggered adoption. This paper does. Finally, we address the geographic clustering of PMCLs. We recommend that future research on opioid policy adoption pay special attention to the geography of the crisis and any parallels in the geography of policy adoption.

## 3. Data and methods

In this section, we detail and motivate the construction of our outcomes. We follow the discussion of the variables with an explanation of our estimation strategy. For reference, Table A2 defines each of the outcomes.

### 3.1. Opioid shipments by destination

To understand how PMCLs affect the dispensing of opioids, we study state opioid shipments by business type: practitioners, chain pharmacies, and local pharmacies. We use data collected as part of the DEA’s Automated Reports and Consolidated Order System (ARCOS), which reports information on opioid shipments. We use a transaction-level data set, which was made public and constructed as part of the National Prescription Opioid Litigation (Evans et al., 2023). The advantage of the transaction-level over the state-level reports is the additional detail regarding business types. The data contain self-reported business activity, which includes several categories of practitioners, chain pharmacies, and local pharmacies.^4^ A common characteristic of pill mills (see Section 2.1) is an on-site pharmacy such that the firm prescribing the opioids is also dispensing them and, thus, receiving the payment. We study opioid shipments to practitioners, which encompasses this behavior.^5^ We will also study shipments to chain pharmacies and local pharmacies separately since pill mills often collude with local pharmacies in return for payments (see Section 2.1).

These data only allow us to observe shipments. It is not recorded whether those opioids were dispensed and used, but they should reflect behavior related to how pill mills operate and we assume that shipments and dispensing are highly correlated (and refer to the results as evidence of “dispensing” behavior). For every transaction, the data include where the opioids were shipped, the date, the total active ingredient weight in grams, and the CDC’s information on strength to convert to MMEs (Centers for Disease Control and Prevention, 2022b). We construct the total MMEs shipped by state, quarter, and type of establishment for 2010–2018.

### 3.2. Measures of opioid prescribing and doctor shopping

For our primary measures, we use administrative prescription drug and medical claims data from the Centers for Medicare & Medicaid Services (CMS) Medicare Part D database from 2010 to 2018 and aggregate variables to the year-quarter-state level (Centers for Medicare & Medicaid Services, 2022). These data include all Medicare beneficiaries enrolled in fee-for-service Medicare. We use the 100% sample. We restrict the sample to beneficiaries who are present in the monthly enrollment files for all three months in a quarter. We identify opioids using the “NDC and Oral MME Conversion File” provided by the CDC (Centers for Disease Control and Prevention, 2022b).^6^ We convert prescriptions to MMEs based on the number of pills and the conversion factors. We only observe filled prescriptions, not prescribing behavior directly. However, we interpret the results as indicative of prescribing behavior. This assumption is supported by the high fill rate among Medicare beneficiaries in part due to drug coverage through Part D (Kim et al., 2016).^7^

Our main measures of prescribing behavior are the number of opioid prescriptions per beneficiary, the share of beneficiaries receiving at least one opioid prescription, total days supplied per beneficiary, and MME doses prescribed per beneficiary-day. These measures reflect general use among the Medicare population.

Given the richness of the data, we construct and examine more nuanced measures to try to isolate inappropriate opioid prescribing. Differentiating between appropriate and inappropriate opioid prescribing is a challenge and yet of central interest when thinking about whether a supply-side regulation can impact overprescribing without troubling impacts on medically-appropriate access. We draw on prior research to inform our selection of suitable proxies for appropriate and inappropriate prescribing behavior (Buchmueller & Carey, 2018; Jena et al., 2014; Zhu et al., 2019) while also relying on qualitative accounts of pill mill operations (Section 2.1) to motivate the construction of additional measures which PMCLs may influence.

We are particularly interested in excessive prescribing over longer time periods. Qualitative evidence (Section 2.1) suggests that pill mills frequently provide 30-day prescriptions and that patients often visit multiple prescribers. This combination of behavior would suggest that we should observe more 30-day prescriptions than calendar months for a given time period. We operationalize this behavior by examining the share of beneficiaries receiving 121+ days supplied in the past 3 months (i.e., more than four 30-day prescriptions in 3 months), and the share of beneficiaries receiving 211+ days prescribed in the past 6 months (i.e., more than seven 30-day prescriptions in 6 months). We do not restrict these measures to only incorporate 30-day prescriptions (i.e., the beneficiaries may receive different prescription lengths that add up to the various thresholds). Prior research has shown that the 211+ day measure is a viable proxy for inappropriate prescribing, as it could indicate drug abuse or diversion (Buchmueller & Carey, 2018). When studying these excessive prescribing measures, we report “per 1,000 beneficiaries.”

We also construct measures that might reflect doctor shopping behavior. Since PMCLs attempt to regulate inappropriate prescribing practices through greater regulation and oversight of pain management clinics, doctor shopping measures are of primary interest to our study, as regulation of providers likely has spillover effects on the care-seeking behavior of patients. Although PMCLs might initially prompt some patients to visit multiple providers when overprescribing clinics shut their doors or change their behavior, the resulting decline in clinics dispensing opioids without adequate patient evaluation may erode both the opportunity and incentive for doctor shopping. This is an empirical question of particular interest to the literature (Chang et al., 2018). We construct the following metrics: overlapping claims, number of prescribers from which a patient received opioid prescriptions in a quarter, and the number of new patient codes per quarter.

For the first measure, an overlapping claim is measured as a second prescription filled for the same ingredient more than a week before the first prescription is expected to finish, given the days supplied of the first prescription. This measure is comparable to metrics studied in the literature (Ali et al., 2019; Buchmueller & Carey, 2018; Jena et al., 2014; Liu et al., 2013). The second measure is a more direct measure of doctor shopping. We count the number of different providers that a beneficiary receives an opioid prescription from during the quarter. Using the number of providers to study doctor shopping is common in the literature (Ali et al., 2019; Delcher et al., 2022; Hall et al., 2008; Jena et al., 2014; Schloff et al., 2004). The third measure, the number of “new patient codes,” is unique from our second measure of doctor shopping as it captures both successful and unsuccessful attempts to receive opioid prescriptions from a doctor (Buchmueller & Carey, 2018).^8^

There are alternative interpretations of our proxies for doctor shopping. Namely, a high quantity of prescribers per patient may be an indication of uncoordinated care, rather than doctor shopping, and most new patients seeking opioid prescriptions are likely not abusing medications. However, we would not expect PMCLs to alter this type of behavior. While imperfect, these proxies are valuable additions to our analysis in providing a broader picture of the potential impacts of PMCLs.

In addition, we study distributional shifts in prescribing by examining the number of opioid prescriptions for 1–7 days, 8–14 days, 15–21 days, and 22+ days. During this time period, 7-days was a common limit for most state laws governing initial opioid prescriptions (Sacks et al., 2021) so we construct measures motivated by this threshold. To the extent that we observe effects on overall opioid prescriptions, it is useful to understand if we observe a reduction in longer opioid prescriptions relative to shorter prescriptions.

Pill mills may—alternatively or in conjunction with long prescriptions—write high-dosage prescriptions. We study the rate of prescriptions with more than 90 MMEs per day (“MEDD” for morphine equivalent daily dosage). The CDC has cautioned against doses higher than 90 MME per day (Dowell, Haegerich, et al., 2016) and this threshold is often used in empirical work to designate high-dose opioid prescriptions (Bradford et al., 2024; Cerdá et al., 2021; Guy, 2017; Zhu et al., 2019).

We are interested not only in the total effects of PMCLs on the Medicare population, but also heterogeneous effects across different subpopulations. We constructed a categorical variable for opioid-naïve status by considering those who had no opioid prescriptions within the previous six months. We conditioned these analyses on beneficiaries who were in the Medicare enrollment files for at least the past six months to ensure that we can appropriately identify all beneficiaries qualified as being opioid naïve.^9^ We divided all outcomes by the number of beneficiaries in a year-quarter-state-category cell to obtain rates.

### 3.3. Pain management clinics and pharmacies

To study how PMCLs affect the supply of pain management clinics and pharmacies, we examine data on state counts of establishments by NAICS-code industry in the Quarterly Census of Employment and Wages (QCEW) and the County Business Patterns (CBP). Using administrative information from unemployment insurance (UI) programs, the QCEW reports the number of establishments at the quarterly level for US counties. The CBP reports employer establishment counts at the annual level for all counties using survey data collected by the Business Registrar. We use the 2010–2018 QCEW. Because CBP are annual data, we use 2009–2018 to ensure a pre-period for all states in our main analysis sample so that we do not have to exclude Florida, which adopted in 2010.

We aggregate CBP data to the state-year level, and the QCEW to the state-year-quarter level and construct measures of establishment counts per 100,000 population. Following Mallatt (2020), we use NAICS codes that are most likely to be involved with pill mills—“All Other Outpatient Centers and Clinics” (or “All Other Outpatient Clinics” hereafter) (NAICS code: 621498), which include pain management clinics, and pharmacies (NAICS code: 446110).

The CBP is known to undercount establishments belonging to multi-unit firms, including chain pharmacies (United States Census Bureau, 2025). On the other hand, the QCEW will undercount establishments, such as pain clinics that are evasive or temporarily in business, if they never register with state UI programs. Pill mills may forego registration with the UI system because registration can expose them to audits and other forms of regulatory scrutiny related to labor laws and licensing. The CBP draws from multiple sources of administrative records (e.g., IRS payroll filings (Form 941), Social Security Administration records, and Census Bureau surveys), and may be more suitable for capturing establishments such as pain clinics that operate briefly or forego UI registration but still file federal tax returns. Due to the complementary merits of each of these data sources, we will study the supply-side impact of PMCLs using both sources.

### 3.4. Overdose deaths

We also examine overdose deaths. We use the National Vital Statistics System (NVSS) Multiple Cause of Death mortality files—the census of deaths in the United States—to study annual overdose deaths from 2010 to 2018 to complement the prescription results (Centers for Disease Control and Prevention, 2022a). We use the restricted version to access state identifiers and categorize overdoses based on the state of residence of the deceased. We code deaths as drug poisonings, which we refer to as “overdoses” throughout this paper, by using ICD-10 external cause of injury codes X40-X44, X60–64, X85, or Y10-Y14. We use drug identification codes for information about the substances found in the body at death. T40.1 indicates poisoning by heroin. T40.2 designates natural and semisynthetic opioids excluding heroin (e.g., oxycodone), T40.3 is methadone, and T40.4 refers to synthetic opioids excluding methadone (e.g., fentanyl, tramadol). Our metric for opioid overdoses includes T40.0-T40.4 plus T40.6. These codes include opium and unspecified narcotics in addition to the categories previously mentioned. We also study “prescription opioid deaths,” defined as those involving T40.2 and T40.3 and “illicit opioid deaths,” defined as deaths involving T40.1 and T40.4. These definitions are imperfect but useful categorizations. We expect PMCLs to have larger impacts on deaths involving prescription opioids. Pill mills prescribe and dispense prescription opioids, including methadone (Department of Justice, 2020a, 2021; Mettler, 2016; Rigg et al., 2010),^10^ so we would be more likely to expect direct effects on deaths involving those substances. We also want to test for the possibility of substitution to illicit opioids as PMCLs could induce some people relying on pill mills to switch to illicit markets. Alternatively, many people who use illicit opioids started with prescription opioids (Powell & Jacobson, 2025) so PMCLs could reduce rates of initiation into opioid dependence and prevent illicit opioid deaths.

There are concerns about missing opioid-related overdoses, such as those coded as involving unspecified drugs (T50.9) (Ruhm, 2017, 2018). We use the procedure documented in Drake and Ruhm (2023) to assign undocumented overdose deaths to opioid-related deaths. Their “preferred regression corrections” involve using decedent characteristics and state fixed effects in a probit regression (estimated separately for each year) to predict opioid-related overdose deaths. We implement this procedure. We impute opioid-related deaths and deaths for our two subcategories: prescription opioid and illicit opioid deaths.

Overdose deaths among the age 65+ population are rare (Hedegaard et al., 2022), and we cannot identify the under age-65 Medicare population in the mortality data. Instead, we study the full population and provide additional results stratified by age. We interpret the prescribing results as indicative of broader changes in prescribing practices and opioid availability in the state which may impact mortality.

### 3.5. Explanatory variables

Details of PMCLs were obtained from the Prescription Drug Abuse Policy System (PDAPS) (Prescription Drug Abuse Policy System, 2023) and confirmed by the authors by manually checking state legislature websites and Justia.com.^11^ A total of nine states (excluding Arizona) passed their first PMCL during our study period. The dates when the PMCLs were first enacted is reported in Table 1.^12^ Geographically, as shown in Fig. A1, the adopting states are located east of the Mississippi River, which has been a crucial dividing line in the opioid crisis (Abouk et al., 2021; O’Donnell, 2017). Our empirical analysis accounts for the non-uniformity of the geographic adoption of the policy and analyzes the importance of this decision for the conclusions of the paper. Louisiana and Texas are “always-treated” during our study time frame as these states adopted their PMCLs before 2010. They are excluded from the sample.

In our fully specified models, we control for fraction of the state population that is White,^13^ fraction of the population ages 65+,^14^ medical marijuana laws with operational and legal dispensaries (Powell et al., 2018), recreational marijuana laws, mandatory access PDMP laws (Horwitz et al., 2021), Medicaid expansion status (Abouk et al., 2021), opioid prescribing guidelines,^15^ and statewide pre-reformulation OxyContin misuse rates interacted with calendar year indicators.^16^ These latter interactions account for the time-specific impacts of exposure to the reformulation of OxyContin and have been shown to strongly predict growth in heroin and synthetic opioid deaths during this time period (Powell & Pacula, 2021). Summary statistics for all variables, prior to the adoption of the first PMCL in our sample, are reported in Table A3. States which adopt PMCLs, on average, had higher rates of opioid prescribing and doctor shopping in the pre-period. Fig. A2–A5 provides trends for all states in our analyses for each outcome, stratified by whether the state adopts a PMCL during the sample period or not. For heavy prescribing and doctor shopping outcomes, in particular, we observe some visual evidence that PMCL and non-PMCL states converge throughout the sample period. The overdose death trends display similar convergence. Our event study analysis will more formally analyze the timing of these relationships with policy adoption.

### 3.6. Empirical specification

We leverage the staggered adoption of PMCLs across states and time to identify their effects on shipments, prescribing, doctor shopping, and overdoses. To account for potential contamination of estimates from treatment effect heterogeneity across states and time, we implement a two-stage difference-in-differences approach (Gardner, 2022).^17^ This approach addresses concerns about staggered adoption and treatment heterogeneity which can bias estimation results in a conventional two-way fixed effects model (Goodman-Bacon, 2021).^18^ In the first stage, we estimate:

(1) $$Y_{\text{st}}(0)=\mu_{\text{s}}+\tau_{t}+X{'}_{st}\Omega +\in_{st},$$

using the subsample of untreated and not-yet-treated units (i.e., *PMCL*_*st*_ = 0). *Y*_*st*_ is the dependent variable for state *s* at time *t*, *μ*_*s*_ are the state fixed effects, *τ*_*t*_ are time fixed effects, *X*_*st*_ are a set of time-varying controls at the state-level (discussed above), and ∈_*st*_ is the idiosyncratic error. *Y*_*st*_(0) represents the untreated outcome and is only observed for untreated observations. Residualizing outcomes gives ${\tilde{Y}}_{st}=Y_{st}-{\hat{Y}}_{st}(0)=Y_{st}-{\hat{\mu }}_{s}-{\hat{\tau }}_{t}-X{'}_{st}\hat{\Omega }$. Using the residualized dependent variable, we estimate the second stage on the full sample:

(2) $${\tilde{Y}}_{st}=\beta \times PMCL_{st}+v_{st},$$

where *PMCL*_*st*_ is a treatment indicator equal to 1 if a state has any PMCL at time *t*. Both stages are population weighted.

When presenting difference-in-differences estimates, we consider coefficient stability by providing estimates in which no covariates are included in the first step, demographic and policy controls are included, and when all control variables are included. We are interested not only in average treatment effects, but also how PMCLs affect outcomes over time. Thus, we extend (2) to include leads and lags of the treatment indicator in the following specification, where time is expressed in quarters:

(3) $${\tilde{Y}}_{st}=\sum_{k=-t_{0}}^{t_{1}}\beta^{k}PMCL_{s,t+k}+v_{st}.$$

This specification permits us to assess the parallel trends assumptions by examining how the outcomes differentially evolve prior to treatment. We will also consider the dynamic effects of the policy since we expect that the impacts of the policy may vary over time. We might expect to observe an immediate effect when the policy is adopted as pain management clinics respond and adapt to the policy. We anticipate that the impacts could grow over time since it takes time to enforce the policy, investigate, gather information, and shut down clinics. Other clinics may then respond to these crackdowns by changing behavior. If PMCLs reduce harmful opioid access, dependence initiation rates may decline, resulting in gradual changes in outcomes. When presenting event study estimates, we show estimates related to 10 quarters before adoption to 20 quarters after adoption.^19^ Two-stage difference-in-differences implicitly normalizes the pre-treatment average to zero. Because the entire pre-period is not shown, some of the event studies presented may not have visually “balanced” pre-treatment estimates around zero. While not all treated units have data for 10 quarters prior to adoption, each pre-treatment estimate has a useful interpretation as the average (adjusted) outcome relative to the mean value for each state prior to treatment.

All of the PMCL adopters are east of the Mississippi River (see Fig. A1, referenced above), which is notable given the geography of the fentanyl crisis. The opioid crisis evolved very differently based on the prevalent types of heroin initially available in illicit markets, which varied based on whether the state was east or west of the Mississippi River (Abouk et al., 2021; Bradford & Lozano-Rojas, 2023; Donahoe & Soliman, 2025; O’Donnell et al., 2017). We can see this by plotting quarterly overdose death rate trends for non-adopters of PMCLs based on whether the state is east or west of the Mississippi River in Fig. A6. Given the non-random geographic adoption of PMCLs, we only use states in the eastern part of the country to construct the comparison group. This decision has little impact on the Medicare prescribing results, but the mortality results are more sensitive. We discuss this issue thoroughly below.

For inference, Gardner (2022) uses a GMM framework (Hansen, 1982) which accounts for the two-stage estimation process. The standard errors are adjusted for dependence at the state level. With treatment heterogeneity, it is known that variance estimates can be conservative (see discussions in Borusyak et al. (2024), de Chaisemartin and d’Haultfoeuille (2024), and Mizushima and Powell (2025)) because cross-unit variation in the treatment effects cannot be isolated from the error terms. Since we rely on event study estimates, it is important to highlight that the pre-treatment estimates should not have conservative confidence intervals since there is no treatment heterogeneity prior to treatment. Before treatment, the confidence intervals reflect the variance of the error term. After treatment, the confidence intervals reflect the variance of the error term and cross-unit treatment heterogeneity, suggesting that we might expect the confidence intervals to expand non-trivially after treatment. We observe this pattern in many of our event studies.

Many of the tables will present “counterfactual outcomes,” the mean counterfactual predictions from the first stage. These predictions permit the calculation of the implied proportional effects corresponding to our treatment effect estimates.

## 4. Results

### 4.1. Total opioid supply by establishment type

**Fig. 1.**
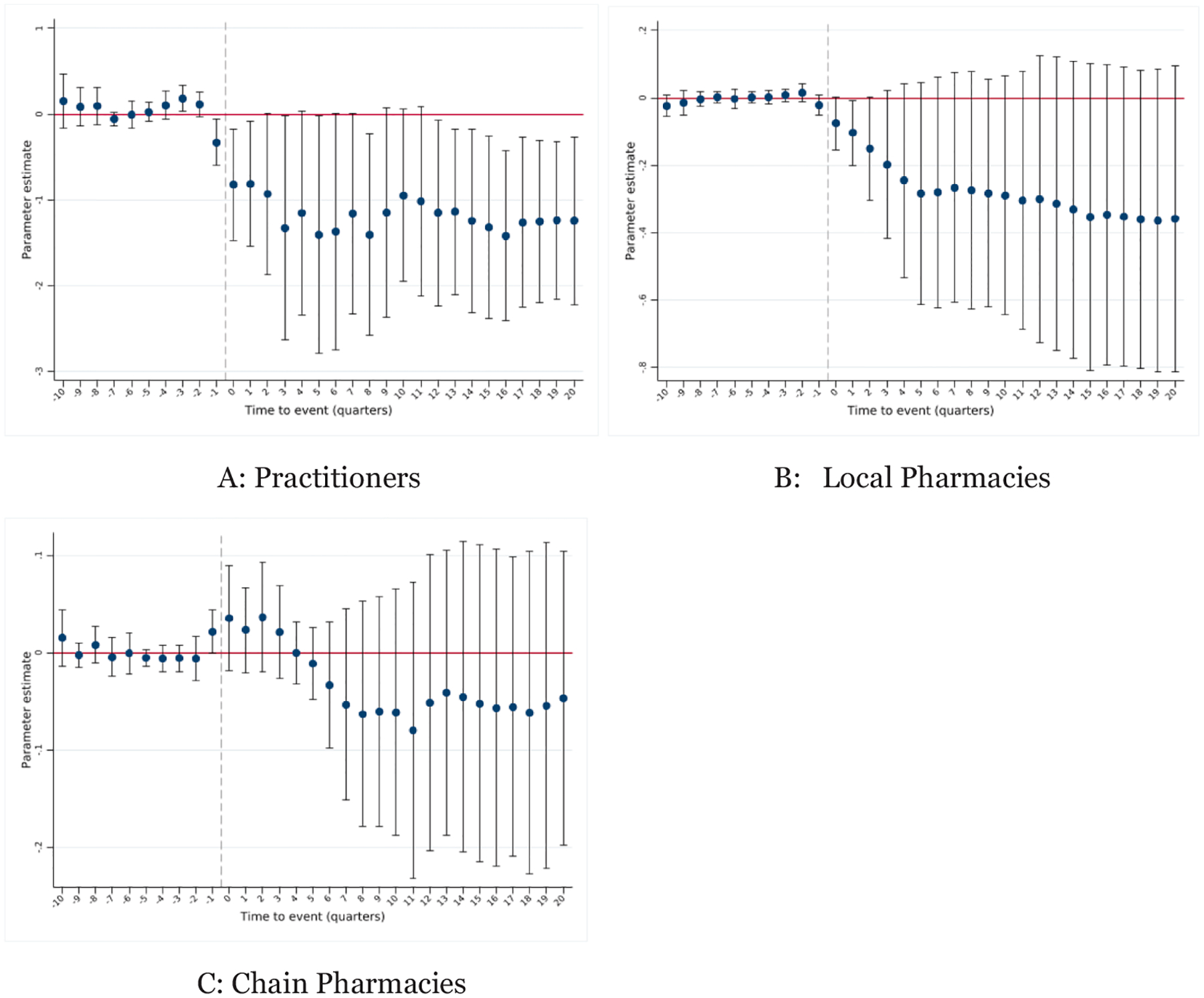
Event Study Estimates For Opioid Distribution by Business Type. Notes: Estimates and 95% confidence intervals (adjusted for state-level clustering) provided. We use two-stage difference-in-differences, weighted by population size. Outcomes are MMEs per capita distributed to each business type and are logged. In the first step, we regress the outcome on state fixed effects, time fixed effects, and covariates using only untreated observations. Only states east of the Mississippi River are included in the analysis. We use the estimates to impute the counterfactuals for the treated units. We regress the difference between the observed outcome and estimated counterfactual on indicators based on quarter-relative-to-adoption (this method does not require normalization). Covariates include share of the state population that is White, share ages 65+, policy variables, and the interaction of the 2004–2009 non-medical OxyContin use rate with year indicators. The policy variables are ACA Medicaid expansion, legal and operational medical marijuana dispensaries, recreational marijuana laws, must-access PDMPs, and opioid prescribing guidelines.

By examining shipments across different establishment types using ARCOS data, we can assess whether PMCLs disrupt the prescribing–dispensing nexus in a way that limits opioid access for misuse. The event study estimates are presented in Fig. 1. We first study the log of opioid shipments (measured in MMEs) per capita to practitioners.^20^ This category should include establishments that both prescribe and dispense opioids. We observe a sharp and striking decline in opioid shipments to these establishments.^21^ The average effect is reported in Table 2 – we estimate that PMCLs reduce opioid shipment quantities to practitioners by 74 percent (using the Panel C estimate),^22^ and the estimate is statistically significant from zero at the 5% level. Shipments directly to practitioners are rare compared to pharmacies, but this large proportional reduction implies a decrease of about 4 MMEs per person in the state.

We also observe a large reduction in shipments to local pharmacies. Qualitative research (Section 2.1) suggests that pill mills may partner with local pharmacies to dispense opioids. Table 2 reports that PMCLs reduce opioid shipments to local pharmacies by 29 percent (statistically significant from zero at the 10% level), which would imply a decline of about 41 MMEs per capita. This relationship is more gradual and grows in magnitude over time (see Fig. 1 Panel B). Finally, chain pharmacies show little net change (an estimated 4 percent decrease, statistically indistinguishable from zero), though the event-study pattern hints at a transitory post-adoption uptick—perhaps reflecting substitution of volume away from practitioners and local pharmacies toward larger chains. Comparing estimates across Panels A-C of Table 2, we observe coefficient stability as the estimates are robust to the inclusion of various controls.

These results suggest that PMCLs deter overall opioid access while targeting business relationships in which pill mills may directly profit from inappropriate opioid prescribing. The large proportional reduction in shipments directly to practitioners suggests that, while small in terms of the share of opioid dispensed to the general population, these shipments may over-represent opioids associated with high rates of misuse.

### 4.2. Opioid prescribing

For our analysis of prescribing behavior, we begin by examining how broader metrics of opioid prescribing respond to PMCLs. Fig. A8 presents event studies for the per-beneficiary rate of receiving any opioid prescription in the quarter, number of opioid prescriptions, opioid days supplied, and MMEs per day prescribed. For each of these outcomes, there is some visual evidence of a slight *increasing* pre-trend in quarters 10 to 4 prior to treatment. However, we observe less differential movement in the year before adoption of the policy. After adoption, there are uniquely (relative to pre-treatment variation) large reductions in all of these outcomes. These reductions persist through the end of the 20 quarters included in the event studies.

We summarize the post-treatment effects in Table 3 and focus on the Panel C estimates. We estimate that PMCLs reduce the share of beneficiaries receiving at least one opioid prescription by 0.5 percentage points, equivalent to a 2 percent decrease, and opioid prescriptions by 3.5 per 100 beneficiaries, equivalent to a 5 percent decrease. We also find that PMCLs reduce days supplied by 0.8 days per beneficiary, implying a 5 percent reduction. We observe a corresponding decline in MMEs per day, equivalent to an 11 percent decrease. These estimates are all statistically different from zero at the 1% level and are generally stable across the different models (Panels A-C), though the addition of the covariates tends to improve the precision of the estimates.

Interestingly, the implied proportional effects are larger for the outcomes incorporating more intensive margin behaviors. The number of prescriptions responds more than whether a beneficiary receives a prescription, while MMEs per day—which captures a broader set of behavioral margins such as days supplied and dosage—responds to the greatest extent. Still, these aggregate measures may obscure changes in more problematic metrics of opioid prescribing. We next study measures of high opioid days supplied over 3 months and over 6 months and high-dosage prescriptions.

### 4.3. Heavy opioid prescribing

Panels A and B in Fig. A9 illustrate the effect of PMCLs on beneficiaries receiving 121+ days per quarter or 211+ days per half-year per 1,000 beneficiaries. We observe little evidence of pre-existing trends, followed by large reductions after PMCL adoption. These patterns are observed both for the share of beneficiaries receiving 121+ days of opioids in a quarter and the share receiving 211+ days in a half-year (starting in January and July).^23^ The estimated effects are summarized in Table 4. We estimate large and statistically significant effects for all outcomes, implying 11–12 percent reductions (Panel C). The estimates are generally stable across the different models (but more precise with covariates). The large proportional reductions, relative to the results for all opioid prescriptions, suggest that PMCLs disproportionately target problematic opioid prescribing.^24^

We study high-dose (>90 MEDD) prescriptions in Fig. A9 Panel C. We observe a sharp decline in these prescriptions after PMCL adoption. Our average effect estimate (Table 4, Panel C) implies that PMCLs reduce high-dose opioid prescriptions by 15 percent, suggesting that this is an important margin.^25^

### 4.4. Doctor shopping measures

Next, we analyze the effect of PMCLs on three measures of potential misuse and doctor shopping: average number of overlapping claims per 1,000 beneficiaries, average number of opioid prescribers per beneficiary, and average number of new patients per beneficiary. Event study graphs (Fig. A10) suggest that PMCLs decrease all of these measures. Referring to the average effects in Table 5 Panel C, PMCLs reduce overlapping claims by 4.8 per 1,000, equivalent to a 20 percent decrease.^26^ PMCLs reduce the number of prescribers per beneficiary by 0.01 (3 percent). Most notably, PMCLs lead to a reduction in the frequency of new patient codes by 14 percent. All of these estimates are statistically different from zero at the 1% level. The estimates suggest meaningful impacts of PMCLs on doctor shopping. Conceptually, it is possible that PMCLs would increase doctor shopping if pill mills substituted for visiting multiple prescribers. Our results, however, confirm anecdotal accounts that pill mills operate in a manner that encourages doctor shopping.

### 4.5. Prescription-Level analysis

We find large changes in days supplied after the adoption of a PMCL. While informative of how a policy may impact overall opioid access, the policy implications are potentially very different depending on how the number of days supplied is reduced. Reductions in the number of opioid prescriptions may be due to blanket reductions in prescriptions; alternatively, they may result from reductions in long prescriptions. We study whether PMCLs were associated with a compositional change in the prescription supply duration.

Fig. A11 reports the effect of PMCLs on the composition of opioid prescriptions. We study the per beneficiary rate of prescriptions with 1–7, 8–14, 15–21, and 22+ days’ supply (the y-axis scales are the same for the four figures). We observe evidence of reductions for all types of prescription lengths. However, the results are stronger—especially in the long-term—for 22+ day prescriptions. The average effects, reported in Table 6, increase monotonically in magnitude as prescription length increases. These findings align with the intended goal of PMCLs to reduce excessive and inappropriate opioid prescribing. While not all long-duration prescriptions are inappropriate, we would expect a policy that reduced inappropriate prescribing to disproportionately impact longer duration prescriptions.

### 4.6. Subgroup analysis

In this section, we consider heterogeneous responses to PMCLs. First, we study differences for the under age-65 (SSDI population) relative to the ages 65+ Medicare population (Table A4, Panels A and B, respectively). The magnitudes are consistently larger for the SSDI population. This is partially because the under age-65 group has more opioid exposure; however, even the implied proportional changes are larger in magnitude. Notably, we estimate that PMCLs reduce opioid days supplied by over 2 days per beneficiary for the SSDI population. We also estimate especially large effect sizes for our measures of high prescribing over time (Columns 5 and 6) and doctor shopping (Columns 8–10).

We next stratify the sample based on prior opioid prescription exposure. We define a beneficiary as opioid-naïve if they did not have an opioid prescription in the previous 6 months. We acknowledge that this definition is itself endogenous to PMCLs since we have found that PMCLs impact rates of receiving opioid prescriptions.^27^

We summarize these findings in Table A4, Panels C and D. On almost every metric, we observe substantially larger level effects for the non-naïve population. Much of this can be attributed to the greater potential scope for policy to impact this population. For example, we estimate that PMCLs reduce the share of non-naïve beneficiaries receiving 211+ days of opioids in a half-year by 6.9 per 1,000, equivalent to a 7 percent decrease. We estimate a reduction of 0.001 per 1,000 (not statistically significant from zero) for the opioid-naïve population, but this estimate implies a 13 percent decrease relative to the counterfactual.

There is interest in policy options that deter opioid initiation, especially problematic use of opioids. We observe strong statistical evidence of reductions in opioid prescriptions among the opioid-naïve population, including 16 percent reductions in MMEs, 24 percent reductions in the share of beneficiaries receiving 121+ days of opioids in a calendar quarter, and 54 percent reductions in high-dosage prescriptions.

### 4.7. Sensitivity analyses

For most outcomes, we observe little evidence of any pre-existing trends, followed by uniquely large reductions after PMCL adoption. The estimates are robust to the inclusion of a large set of demographic and policy controls. While the fentanyl crisis has disproportionately affected some states more than others, our findings are robust to accounting for each state’s predisposition to the fentanyl crisis.

As a sensitivity test, we further restrict our sample with the motivation of avoiding states in the northeast and/or coastal states due to concerns that they also tended to have different experiences with the illicit opioid crisis. We only include PMCL-adopters plus Michigan, Indiana, and Illinois. This test uses adopting states as controls prior to adoption plus three non-adopting states. The results, shown in Table A5, remain consistent with our main findings: despite increased noise due to the smaller comparison set of states, we still estimate large effects on aggregate prescribing measures and strong evidence of doctor shopping effects. This confirms that our core conclusions are not driven by the choice of comparison states, although the full set of comparators in our primary analysis provides greater statistical power.

Florida adopted a PMCL while simultaneously using other policy and enforcement mechanisms to address the prevalence of pill mills in their state. We test whether Florida is driving our results by excluding Florida from our analyses and presenting the results in Table A6, replicating Panel C of Tables 3–5. We generally find that the exclusion of Florida does not affect the main conclusions. Some magnitudes decrease (e.g., MMEs), though we still find strong evidence of effects on heavy prescribing over longer time periods and other outcomes.

More broadly, we focus on PMCLs in this paper, but our time period represents an especially active period of federal and state policy adoption to address opioid-related harms. This is problematic if PMCL adoption is often linked—intentionally or unintentionally—with the adoption of other policies. While we control for many of these policies and find that the inclusion of these controls has little effect on the main conclusions of the paper, it is difficult to isolate the impacts of multiple policies which are adopted at different times and may have heterogeneous treatment effects. To consider the possible confounding nature of other policies, we study the relationship between PMCL adoption and other policy adoption by estimating our main specification but including each policy as the outcome. We study a broader set of policies than those included in our models. The results are presented in Table A7.

We find evidence that PMCL adopters are less likely to adopt e-prescribing mandates, which have been found to reduce opioid use and deaths (Abouk & Powell, 2021), or implement prescribing limits, which the literature suggests have no influence on deaths (Abouk & Powell, 2021) but with some evidence of changing prescribing behavior (Allen et al., 2024; Sacks et al., 2021). This would attenuate our estimates. PMCL states are less likely to adopt medical cannabis laws, which we define as states with legal and operational medical marijuana dispensaries, which have been shown to reduce opioid prescribing and overdose deaths (Bradford et al., 2018; Powell et al., 2018; Smith, 2020).^28^

We also find that PMCL adoption is positively associated with the implementation of opioid prescribing guidelines, which would likely also lead to declines in opioid prescribing measures. However, we previously found that PMCLs primarily affect dispensing by practitioners and through local pharmacies with less evidence of any impact on chain pharmacies. Opioid prescribing guidelines target prescriptions for acute pain and prescribing in emergency departments. These prescriptions would typically be filled in pharmacies, and we would expect to observe reductions in opioids for all types of pharmacies, including chain pharmacies. The reductions in opioids observed in Fig. 1 above are more consistent with a policy targeting establishments with suspect agreements with local opioid dispensers.

Overall, we conclude that it is unlikely that our estimates are driven by concurrent policy adoption. Related, we find little evidence of negative lead effects despite some of these policies being adopted prior to the PMCLs. Instead, we generally estimate immediate PMCL effects, but none of the policies studied in this section were adopted in the same quarter as the PMCL or in the quarter before the PMCL was implemented.^29^

### 4.8. Mechanisms – pain clinic and pharmacy establishments

Our conceptual framework outlines that PMCLs may impact opioid prescribing metrics through closures of pill mills or through behavior changes among existing pain clinics. Here, we directly study the impacts of PMCLs on “all other outpatient clinics,” a useful proxy for pain management clinics, and on pharmacies. We replicate the analysis in Mallatt (2020) with updated data and using two-stage difference-in-differences. Following Mallatt (2020), the outcomes are the log of the number of establishments per capita.

We find little evidence of systematic trends in per-capita pain clinics prior to PMCL adoption followed by a decline which gradually grows in magnitude over time (see Fig. A12). The estimates starting in quarter 20 post-adoption are statistically different from zero at the 5% level (though our event study figures only present results for all quarters leading up to and including quarter 20). Our average effect estimate, reported in Table A8, suggests that PMCLs reduce the prevalence of pain clinics by over 14 percent (statistically significant at the 10% level). There is little evidence of immediate effects, though we estimated immediate impacts on prescribing outcomes. This suggests that PMCLs either alter the prescribing behavior of existing clinics or facilitate the gradual entry of pain management clinics with more appropriate prescribing practices as pill mills exit the market.

Panel B in Fig. A12 provides the results for pharmacies. We observe some evidence of pharmacy exit with larger estimated reductions in later time periods. We estimate an average effect of −5.4 percent, not statistically different from zero. This result is sensitive to the inclusion of controls – without controls, the estimate implies a 9 percent reduction and is statistically significant from zero. Overall, our estimated effect is reasonably close to parallel estimates in Mallatt (2020) and Burton and Churchill (2025).^30^

We analyze the same outcomes using the CBP. The pattern of estimates for “all other outpatient clinics” is similar (see Fig. A12, Panel C). We estimate small short-term reductions followed by larger long-term reductions (statistically significant four years post-adoption). The average effect implies a 3 percent reduction in these clinics (not statistically different from zero). There is less evidence of an effect on pharmacies in the CBP.

Overall, it is difficult to observe pain management clinics in available data resources. To the extent that we can study them, we find limited but suggestive evidence that PMCLs contribute to the closure of pill mills. The results suggest that any short-term prescribing effects and dispensing effects are due to behavioral changes in existing pain management clinics while also indicating that PMCLs could have longer-term impacts through closures.^31^

## 5. Consequences on overdose mortality

### 5.1. Estimating PMCL impacts on opioid-related overdose deaths

We found evidence of a decline in opioid prescriptions among the Medicare population following PMCL adoption, especially measures related to overprescribing and doctor shopping. We are interested in the mortality consequences of these prescribing changes, as mortality outcomes provide a window into potential substitution to illicit markets. We cannot select on Medicare beneficiaries because Medicare eligibility is not reported in the NVSS. Instead, we report results for the total population, for those aged 18–64, and for those aged 65 and older.

**Fig. 2.**
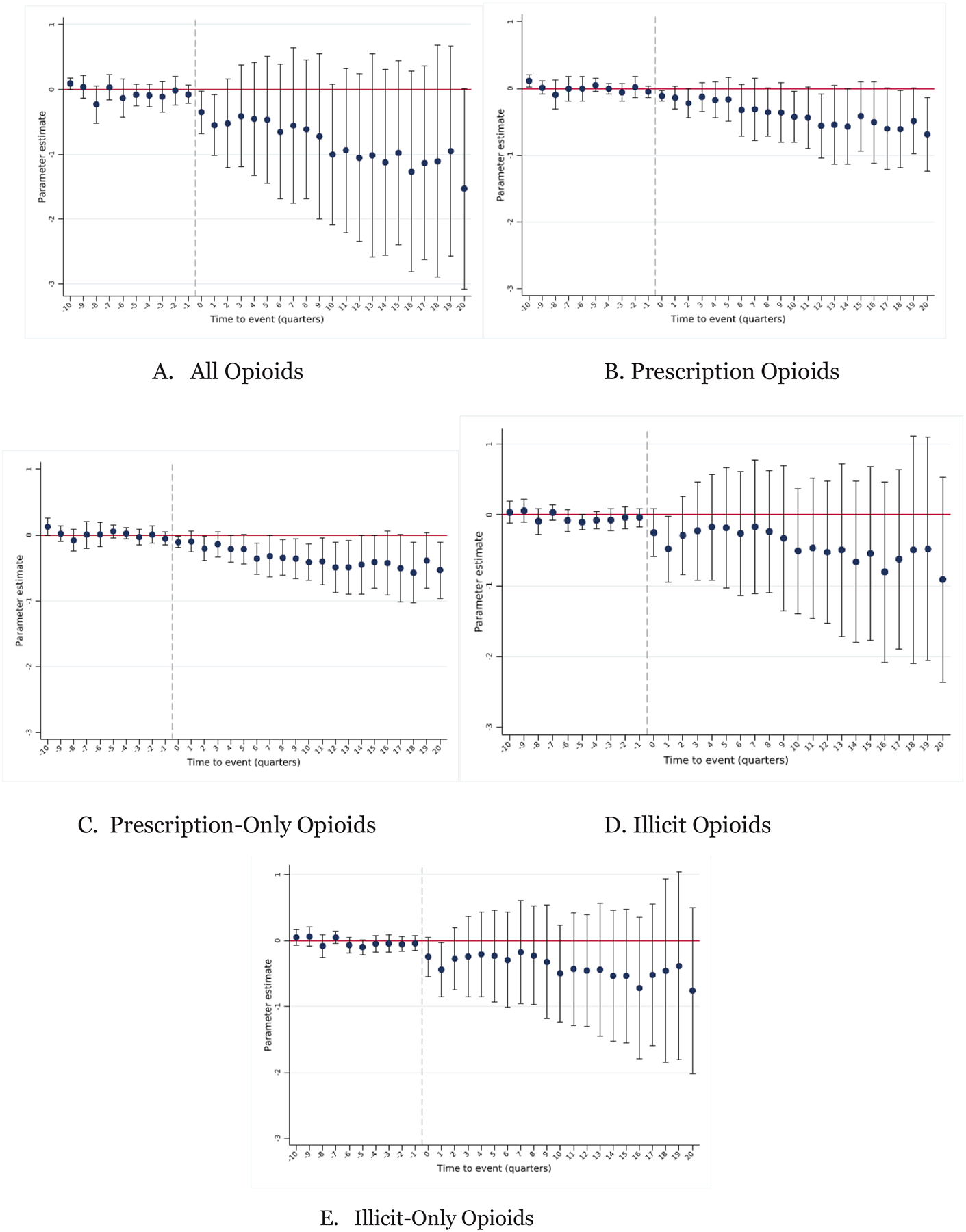
Event Study Estimates for Overdose Deaths (per 100,000). Notes: Estimates and 95% confidence intervals (adjusted for state-level clustering) provided. Model is same as the Table 7, Panel C model. See Table 7 notes for further details. Estimates refer to the full population.

Table 7 presents the main estimates. The point estimates are generally similar across Panels A-C though the controls for these outcomes tend to reduce the precision of the estimates. We estimate that PMCLs reduce opioid-related overdose deaths by 0.9 deaths per 100,000 people. This estimate is statistically significant from zero at the 10% level in Panel A but not statistically significant in Panel C. The effect size for prescription opioid deaths is about half of the overall effect, statistically significant at the 5% level in each model. The estimated effects for illicit opioids (Column 4) are also negative, though not statistically different from zero. We provide the corresponding event studies in Fig. 2. The impacts emerge soon after PMCL adoption for all opioids (Panel A) and for prescription opioids (Panel B), and the effects grow over time. This pattern is consistent with PMCLs removing a source of overdose deaths in the short-term and reducing dependence initiation with longer-term consequences on mortality.

Table 7 and Fig. 2 also include results in which the outcome is overdose deaths involving prescription opioids but no illicit opioids (Column 3) and deaths involving illicit opioid but no prescription opioids (Column 5). These results are provided to test whether mixing of prescription and illicit opioids is driving the relative importance of each type of overdose. We find that the estimates are similar when prescription (illicit) opioids are excluded. We include mortality results without the Drake and Ruhm (2023) correction in Table A9. The point estimates are generally smaller in magnitude, consistent with the unadjusted rates “missing” deaths impacted by the policy. The implied proportional effects are, however, similar.

The main effects in Table 7 imply a reduction in prescription opioid overdose deaths by 21.5 percent. This pronounced effect suggests that these policies are especially effective in targeting problematic opioid access/dispensing in a way that our prescribing metrics do not perfectly encapsulate. Notably, we find the strongest evidence for reductions in deaths involving prescription opioids, which are more directly targeted by PMCLs, though the point estimates for deaths involving prescription opioids and illicit opioids are similar. One concern with the “illicit opioid” designation here is that we include all synthetic opioids in this category, even though there are many synthetic opioids which are prescribed, potentially conflating those definitions. While PMCLs may have prompted some to shift toward illicit opioids, they may also have prevented others from initiating opioid use, leading to an overall decline in opioid-related mortality. The point estimates suggest that this latter mechanism dominated.

Table A10 provides the same results disaggregated by more specific opioid types. We find strong evidence of reductions in natural/semisynthetic opioids, consistent with our estimates of decreases in inappropriate opioid prescribing. We also estimate statistically significant reductions in methadone, which is notable given that qualitative evidence (see Section 3.4) has found that pill mills dispense large volumes of methadone.

Table A11 reports mortality results by age group. The magnitudes are generally larger for the 18–64 population. We observe smaller effects for the 65+ population, and we can never statistically reject that there was no change in mortality for this group.

### 5.2. Comparisons to the literature

The most comparable study to ours is Cerdá et al. (2021), which finds evidence of causal *increases* in deaths involving heroin and/or synthetic opioids. There are many methodological differences between our study and Cerdá et al. (2021) (see Section 2.5). We focus here on the role of the geography of PMCL adoption and fentanyl penetration, both of which disproportionately affected states east of the Mississippi River (O’Donnell et al., 2017). In Panel A of Table A12, we repeat our main mortality results. Panel B presents the results when we include states in the west as part of the comparison group.^32^ Including states in the west, we find evidence—similar to the conclusions reached in Cerdá et al. (2021)—that PMCLs led to a shift to illicit opioids, increasing deaths involving heroin or synthetic opioids. The estimates suggest that PMCLs increase illicit opioid deaths by 10 percent, though the estimate is not statistically significant from zero.

One concern with the sensitivity of the mortality results to accounting for geographic-specific time shocks is that it may suggest further sensitivity to factors that we are not including in our model. However, this is unlikely to be the case given the event study results. We observe substantial east/west differences in mortality among non-PMCL adopters (Fig. A5). If there were similar within-region heterogeneity, then this would appear as pre-treatment shocks in the event studies given that states adopted PMCLs over time.

The prescribing results are less sensitive to accounting for geographic-specific shocks, which is not surprising given that the intended purpose of our regional restriction is to control for changes in the illicit market. We compare the Medicare prescribing results with and without the states in the west in Table A13. In general, the estimates are similar.

## 6. Conclusion and discussion

We find that PMCLs are associated with decreases in opioid shipments to practitioners dispensing opioids and local pharmacies—entities most likely to be associated with pill mills. This result suggests that PMCLs accomplish their main goal of diminishing the relationship between opioid prescribing and profit incentives which encourage inappropriate prescribing behavior.

We observe concurrent decreases in total opioid prescribing, over-prescribing, and doctor shopping among Medicare beneficiaries. In general, we estimate larger proportional (to baseline) effects for measures associated with more problematic opioid-related behavior. Most glaringly, we estimate mortality reductions which are large relative to the decreases in prescribing and even measures of problematic prescribing. This pattern is consistent with PMCLs targeting inappropriate opioid access, which cannot be perfectly measured in claims data. The decreases in total prescribing and overprescribing are driven primarily by non-naïve Medicare beneficiaries and the SSDI population. However, we also observe reductions in prescribing to opioid-naïve beneficiaries, suggesting that PMCLs may potentially reduce opioid dependence initiation with longer-term consequences on opioid misuse and doctor shopping.

Supply-side regulations often have unintended negative consequences on patients with opioid use disorder by inducing substitution from prescription to illicit drug use (Alpert et al., 2018; Beheshti, 2019; Mallatt, 2022; Meinhofer, 2018; Powell et al., 2019). Our sample period is during the illicit opioid crisis and other supply-side interventions during this time period have been shown to encourage substitution to illicit opioid markets (Kim, 2021). Such laws may induce sharp decreases in opioid access without adequate treatment alternatives for patients dependent on opioids. Unlike prior research, our findings on mortality suggest that PMCLs do not suffer from these unintended consequences or that the mortality reductions due to PMCLs outweigh the harms associated with such transitions to illicit opioid markets.

Methodologically, our study also shows the importance of considering the geography of the fentanyl crisis when studying PMCLs. Given the temporal and geographic concordance between the growing fentanyl crisis east of the Mississippi River and PMCLs, including states west of the Mississippi River in estimates would produce biased estimates that could lead one to incorrectly infer that illicit drug mortality increased due to PMCLs. Such policy “clustering” is the norm when studying opioid-specific policies and these geographic effects should be accounted for in research on the opioid crisis.

Our results suggest that PMCLs may operate with a lag. Opioid shipments to practitioners react immediately, possibly suggesting that these were the clearest cases of inappropriate opioid dispensing behavior. However, shipments to local pharmacies were increasingly affected through the sample period, and the prescribing and mortality impacts also grew over time. There are some potential reasons for this. First, it may take time after PMCLs are enacted for authorities to gather the evidence resulting from the laws and investigate potential pill mills. To the extent that PMCLs work by building a paper trail for investigators (as discussed in Section 2.2), we would expect lagged effects. Second, the deterrent effect of PMCLs may take time if such closures occur with a delay. For both of these points, the evidence in Section 4.8 suggests that any effect on closures is lagged. Third, subclauses of PMCLs typically increase in scope and oversight authority over time. For example, facility inspections were introduced with a two-year lag in Mississippi and Ohio. Fourth, PMCLs may affect opioid dependence rates. By curbing harmful prescribing practices, PMCLs may help prevent the onset of opioid dependence, which typically involves escalating use over time. As a result, the adoption of PMCLs is likely to reduce the prevalence of harmful opioid prescribing practices more in the long term than in the short term.

We note a few important limitations of our study. First, we only observe prescribed and legally purchased opioids. We are unable to identify the receipt of opioids obtained through other means. We also do not observe prescriptions that were not paid by Medicare, either because the patient did not pick up the prescription or because they paid in cash, implying that we do not perfectly measure prescribing or consumption behavior. To the extent that PMCLs cause patients to begin paying in cash, some of the effects on prescribing outcomes may not be true indicators of changes in opioid consumption. However, the mortality effects and the impacts on shipments to practitioners suggest that problematic opioid access is decreasing. Second, our concurrent policy adoption test reveals that PMCL states are less likely to adopt prescribing limit laws and medical cannabis laws compared to control states and more likely to implement opioid prescribing guidelines. To the extent that prescribing limit laws and medical cannabis laws decrease opioid prescribing, our results will be biased downward. The guidelines seem unlikely to be driving the effects given that we do not observe more uniform reductions in dispensing across establishment types. Finally, we note that PMCLs are only one policy lever for decreasing inappropriate prescribing by providers. The second and third waves of the epidemic are driven by rises in overdoses due to heroin and synthetic opioids. While PMCLs have an important role in regulating quasi-legal opioid markets, it may be difficult to extrapolate our findings to the evolving crisis.

Despite these limitations, our study has important implications for policy. The results suggest that policies which target the underlying quality of care for pain management rather than imposing blanket restrictions or hassles on prescribing might be more effective at reducing harmful prescribing while also minimizing the risk of substitution into illicit drug use. Unlike other supply-side regulations, PMCLs appear to target high-risk prescribing with less effect on legitimate pain management access (Rutkow et al., 2017). More generally, there appears to be a role for regulatory oversight and enforcement in quasi-legal settings to address some of the harms of the opioid crisis.

PMCLs are still not widely adopted, but this paper suggests that they carry promise even as the opioid crisis transitions further to illicit opioids. PMCLs are costly to enforce, requiring substantial resources from law enforcement and state medical and health agencies. These costs partially explain their limited adoption across the country (Rutkow et al., 2017). In addition, physicians are often opposed to the additional regulations imposed by such legislation and actively lobby against their adoption (e.g., California Medical Association (2023)). The results of this paper imply that these costs are likely worth paying.

## Supplementary Material

Appendix

## Figures and Tables

**Table 1:** Pain management clinic law adoption by state.

State (Policy Date)	Clinic and Practice Oversight	Qualifications for Practitioners	Qualifications for Ownership
Alabama (5/8/2013)	✔	✔	✔
Arizona (4/26/2018)			
Florida (10/1/2010)	✔	✔	✔
Georgia (7/1/2013)	✔	✔	✔
Kentucky (7/20/2012)	✔	✔	✔
Louisiana (7/11/2005)	✔	✔	✔
Mississippi (4/24/2011)	✔	✔	✔
Ohio (5/20/2011)	✔	✔	✔
Tennessee (5/30/2011)	✔		✔
Texas (9/1/2009)	✔	✔	✔
West Virginia (6/8/2012)	✔	✔	✔
Wisconsin (3/19/2016)	✔		✔

*Notes:* We used PDAPS and our own legal research to categorize PMCL policies. Arizona is excluded from the analysis because its policy did not include any penalties or inspections until 2019. Louisiana and Texas are also excluded because they implemented their policies prior to our sample period. See Table A1 for more detailed information on these policies.

**Table 2:** Difference-in-differences estimates for opioid shipments.

	Panel A: State and Time Fixed Effects		
	*Outcome* = log*(MME Per Capita)*		
	(1)	(2)	(3)
	Practitioners	Non-Chain Pharmacies	Chain Pharmacies
PMCL	−1.385** (0.549)	−0.321 (0.202)	−0.045 (0.056)
	**Panel B: Demographic and Policy Controls**		
PMCL	−1.350** (0.557)	−0.341 (0.209)	−0.045 (0.060)
	**Panel C: OxyContin Misuse**		
PMCL	−1.334** (0.548)	−0.344* (0.209)	−0.046 (0.059)

Notes:

* 10%,

** 5%,

*** 1% statistical significance. Standard errors (adjusted for state-level clustering) provided. Outcomes are derived from the transaction-level ARCOS for 2010–2018. We use two-stage difference-in-differences, weighted by state population. In the first step, we regress the outcome on state fixed effects, time fixed effects, and covariates using only untreated observations. Only states east of the Mississippi River are included in the analysis. We use the estimates to impute the counterfactuals for the treated units. We regress the difference between the observed outcome and estimated counterfactual on whether the state had enacted a PMCL. “Demographic and Policy Controls” include share of the state population that is White, share of Medicare beneficiaries ages 65+, and policy variables. The policy variables are ACA Medicaid expansion, legal and operational medical marijuana dispensaries, recreational marijuana laws, must-access PDMPs, and opioid prescribing guidelines. Panel C includes controls for the interaction of the 2004–2009 non-medical OxyContin use rate with year indicators.

**Table 3:** Difference-in-Differences Estimates for Medicare Prescribing Outcomes.

	Panel A: State and Time Fixed Effects			
	(1)	(2)	(3)	(4)
	Any Opioids	Number of Prescriptions	Days Supply	MME
PMCL	−0.006 (0.004)	−0.031 (0.021)	−0.577 (0.423)	−0.815*** (0.284)
Counterfactual Mean	0.253	0.673	15.111	8.634
Implied Percent Change	−2.402	−4.569	−3.815	−9.439
	**Panel B: + Demographic and Policy Controls**			
PMCL	−0.006** (0.002)	−0.035*** (0.010)	−0.772*** (0.238)	−0.925** (0.422)
Counterfactual Mean	0.252	0.677	15.306	8.744
Implied Percent Change	−2.207	−5.187	−5.041	−10.575
	**Panel C: + OxyContin Misuse**			
PMCL	−0.005*** (0.002)	−0.035*** (0.008)	−0.766*** (0.213)	−0.928** (0.411)
Counterfactual Mean	0.252	0.676	15.300	8.748
Implied Percent Change	−2.147	−5.111	−5.004	−10.612

Notes:

* 10%,

** 5%,

*** 1% statistical significance. Standard errors (adjusted for state-level clustering) provided. All outcomes are per-beneficiary. We use two-stage difference-in-differences, weighted by the number of beneficiaries. Only states east of the Mississippi River are included in the analysis. In the first step, we regress the outcome on state fixed effects, time fixed effects, and covariates using only untreated observations. We use the estimates to impute the counterfactuals for the treated units. We regress the difference between the observed outcome and estimated counterfactual on whether the state had enacted a PMCL. “Demographic and Policy Controls” include share of the state population that is White, share of Medicare beneficiaries ages 65+, and policy variables. The policy variables are ACA Medicaid expansion, legal and operational medical marijuana dispensaries, recreational marijuana laws, must-access PDMPs, and opioid prescribing guidelines. Panel C includes controls for the interaction of the 2004–2009 non-medical OxyContin use rate with year indicators. The “Counterfactual Mean” is the mean of the outcome for all treated observations after subtracting off the estimated treatment effect. “Percent Change” is the implied percent change of the estimate given the counterfactual mean.

**Table 4:** Difference-in-Differences Estimates for Heavy Prescribing Outcomes.

	Panel A: State and Time Fixed Effects		
	(1)	(2)	(3)
	121+ Days Per Quarter (per 1,000)	Days Per Half-Year (per 1,000)	Prescriptions > 90 MEDD (per 1,000)
PMCL	−1.843 (1.550)	−2.508 (2.003)	−10.105** (4.810)
Counterfactual Mean	23.407	28.768	70.356
Implied Percent Change	−7.873	−8.718	−14.363
	**Panel B: + Demographic and Policy Controls**		
PMCL	−2.682*** (0.679)	−3.560*** (0.843)	−10.375** (4.822)
Counterfactual Mean	24.246	29.819	70.625
Implied Percent Change	−11.060	−11.938	−14.690
	**Panel C: + OxyContin Misuse**		
PMCL	−2.664*** (0.565)	−3.533*** (0.661)	−10.570** (4.269)
Counterfactual Mean	24.228	29.793	70.821
Implied Percent Change	−10.994	−11.859	−14.925

Notes:

* 10 %,

** 5%,

*** 1% statistical significance. Standard errors (adjusted for state-level clustering) provided. We use two-stage difference-in-differences, weighted by the number of beneficiaries. Only states east of the Mississippi River are included in the analysis. In the first step, we regress the outcome on state fixed effects, time fixed effects, and covariates using only untreated observations. We use the estimates to impute the counterfactuals for the treated units. We regress the difference between the observed outcome and estimated counterfactual on whether the state had enacted a PMCL. “Demographic and Policy Controls” include share of the state population that is White, share of Medicare beneficiaries ages 65+, and policy variables. The policy variables are ACA Medicaid expansion, legal and operational medical marijuana dispensaries, recreational marijuana laws, must-access PDMPs, and opioid prescribing guidelines. Panel C includes controls for the interaction of the 2004–2009 non-medical OxyContin use rate with year indicators. The “Counterfactual Mean” is the mean of the outcome for all treated observations after subtracting off the estimated treatment effect. “Percent Change” is the implied percent change of the estimate given the counterfactual mean. MEDD = morphine equivalent daily dosage (=MMEs per days supplied).

**Table 5:** Difference-in-Differences Estimates for Doctor Shopping Outcomes.

	Panel A: State and Time Fixed Effects		
	(1)	(2)	(3)
	Overlapping Prescriptions (per 1,000)	Number of Prescribers (per beneficiary)	New Patients (per beneficiary)
PMCL	−3.676** (1.536)	−0.012* (0.007)	−0.017** (0.008)
Counterfactual Mean	22.510	0.328	0.151
Implied Percent Change	−16.333	−3.584	−11.232
	**Panel B: + Demographic and Policy Controls**		
PMCL	−4.781*** (1.175)	−0.011*** (0.003)	−0.022*** (0.004)
Counterfactual Mean	23.614	0.327	0.156
Implied Percent Change	−20.247	−3.295	−14.254
	**Panel C: + OxyContin Misuse**		
PMCL	−4.818*** (1.055)	−0.011*** (0.002)	−0.022*** (0.004)
Counterfactual Mean	23.651	0.327	0.156
Implied Percent Change	−20.370	−3.267	−14.131

Notes:

* 10%,

** 5%,

*** 1% statistical significance. Standard errors (adjusted for state-level clustering) provided. We use two-stage difference-in-differences, weighted by the number of beneficiaries. Only states east of the Mississippi River are included in the analysis. In the first step, we regress the outcome on state fixed effects, time fixed effects, and covariates using only untreated observations. We use the estimates to impute the counterfactuals for the treated units. We regress the difference between the observed outcome and estimated counterfactual on whether the state had enacted a PMCL. “Demographic and Policy Controls” include share of the state population that is White, share of Medicare beneficiaries ages 65+, and policy variables. The policy variables are ACA Medicaid expansion, legal and operational medical marijuana dispensaries, recreational marijuana laws, must-access PDMPs, and opioid prescribing guidelines. Panel C includes controls for the interaction of the 2004–2009 non-medical OxyContin use rate with year indicators. The “Counterfactual Mean” is the mean of the outcome for all treated observations after subtracting off the estimated treatment effect. “Percent Change” is the implied percent change of the estimate given the counterfactual mean.

**Table 6:** Difference-in-Differences Estimates for Prescriptions by Days Supplied.

	Panel A: State and Time Fixed Effects			
	(1)	(2)	(3)	(4)
	Prescription Length (per Beneficiary)			
	1–7 Days	8–14 Days	15–21 Days	22+ Days
PMCL	−0.005 (0.008)	−0.006* (0.003)	−0.009*** (0.003)	−0.011 (0.011)
Counterfactual Mean	0.125	0.063	0.071	0.413
Implied Percent Change	−4.013	−9.162	−13.064	−2.570
	**Panel B: + Demographic and Policy Controls**			
PMCL	−0.005 (0.008)	−0.005** (0.002)	−0.008*** (0.003)	−0.017** (0.008)
Counterfactual Mean	0.125	0.063	0.070	0.419
Implied Percent Change	−4.107	−8.259	−11.699	−3.961
	**Panel C: + OxyContin Misuse**			
PMCL	−0.005 (0.008)	−0.005** (0.002)	−0.008*** (0.002)	−0.017** (0.008)
Counterfactual Mean	0.125	0.062	0.070	0.419
Implied Percent Change	−3.995	−7.962	−11.574	−3.940

Notes:

* 10%,

** 5%,

*** 1% statistical significance. Standard errors (adjusted for state-level clustering) provided. Outcomes are the number of prescriptions for the specified number of days supplied (per beneficiary). We use two-stage difference-in-differences, weighted by the number of beneficiaries. Only states east of the Mississippi River are included in the analysis. In the first step, we regress the outcome on state fixed effects, time fixed effects, and covariates using only untreated observations. We use the estimates to impute the counterfactuals for the treated units. We regress the difference between the observed outcome and estimated counterfactual on whether the state had enacted a PMCL. “Demographic and Policy Controls” include share of the state population that is White, share of Medicare beneficiaries ages 65+, and policy variables. The policy variables are ACA Medicaid expansion, legal and operational medical marijuana dispensaries, recreational marijuana laws, must-access PDMPs, and opioid prescribing guidelines. Panel C includes controls for the interaction of the 2004–2009 non-medical OxyContin use rate with year indicators. The “Counterfactual Mean” is the mean of the outcome for all treated observations after subtracting off the estimated treatment effect. “Percent Change” is the implied percent change of the estimate given the counterfactual mean.

**Table 7:** Difference-in-Differences Estimates for Overdose Deaths per 100,000.

	Panel A: State and Time Fixed Effects				
	(1)	(2)	(3)	(4)	(5)
	Opioids	Prescription Opioids	Prescription Opioids Only	Illicit Opioids	Illicit Opioids Only
PMCL	−0.933* (0.495)	−0.570*** (0.181)	−0.496*** (0.170)	−0.505 (0.428)	−0.430 (0.388)
Counterfactual Mean	4.935	2.376	1.800	3.086	2.509
Implied Percent Change	−18.898	−23.982	−27.556	−16.368	−17.153
	**Panel B: + Demographic and Policy Controls**				
PMCL	−0.936 (0.757)	−0.491** (0.230)	−0.435*** (0.161)	−0.469 (0.653)	−0.411 (0.568)
Counterfactual Mean	4.938	2.298	1.739	3.050	2.490
Implied Percent Change	−18.950	−21.386	−25.001	−15.380	−16.515
	**Panel C: + OxyContin Misuse**				
PMCL	−0.949 (0.769)	−0.495** (0.237)	−0.435*** (0.162)	−0.478 (0.658)	−0.416 (0.570)
Counterfactual Mean	4.952	2.302	1.739	3.059	2.495
Implied Percent Change	−19.173	−21.521	−25.010	−15.629	−16.692

Notes:

* 10%,

** 5%,

*** 1% statistical significance. Standard errors (adjusted for state-level clustering) provided. We use two-stage difference-in-differences, weighted by population size. Only states east of the Mississippi River are included in the analysis. In the first step, we regress the outcome on state fixed effects, time fixed effects, and covariates using only untreated observations. We use the estimates to impute the counterfactuals for the treated units. We regress the difference between the observed outcome and estimated counterfactual on whether the state had enacted a PMCL. “Demographic and Policy Controls” include share of the state population that is White, share of population ages 65+, and policy variables. The policy variables are ACA Medicaid expansion, legal and operational medical marijuana dispensaries, recreational marijuana laws, must-access PDMPs, and opioid prescribing guidelines. Panel C includes controls for the interaction of the 2004–2009 non-medical OxyContin use rate with year indicators. The “Counterfactual Mean” is the mean of the outcome for all treated observations after subtracting off the estimated treatment effect. “Percent Change” is the implied percent change of the estimate given the counterfactual mean.

## Data Availability

The authors do not have permission to share data.

## Footnotes

1 See Department of Justice (2019a, 2019b, 2020a, 2020b, 2021, 2023, 2025a, 2025b, 2025c) for a small subset of recent cases uncovering large pill mills.

2 A separate set of studies investigate the effect of PMCLs on suicides and birth/infant outcomes (Fernandez & Jayawardhana, 2022; Ziedan & Kaestner, 2024).

3 Dowell, Zhang, et al. (2016) studied the combined effect of PDMPs and PMCLs for the 2006–2013 period and estimated an 8 percent decline in opioid prescriptions and a 12 percent decline in fatal opioid overdose deaths.

4 There are no official guidelines to self-determine whether a business is a chain pharmacy or a non-chain (“retail” or “local”) pharmacy, but we consider these categories as useful designations for our analyses. Chain pharmacies include Kroger, CVS, Rite Aid, etc.

5 We aggregate shipments from several different categories of practitioners for this analysis.

6 This file was discontinued in 2022. Additional information on these data can be found here: https://www.cdc.gov/opioids/data-resources/index.html.

7 Kim et al. (2016) report that opioid fill rates after emergency department discharges are two times higher among patients with Medicare, Medicaid, or state indigent assistance compared to those without public health insurance.

8 A new patient code has a HCPCS code of 99201, 99202, 99203, 99204, or 99,205 in the outpatient or physician (carrier) medical claims.

9 We used 2009 data to make this designation for the 2010 sample.

10 While methadone is used to treat people with opioid dependence, it has often been diverted and misused throughout the opioid crisis (Cicero & Inciardi, 2005; Inciardi et al., 2007).

11 The dates of these policies coincide with those used in Cerdá et al. (2021) with the exception that we exclude Arizona from the analysis for reasons discussed later.

12 We use the effective date, though some state policies delayed their enforcement provisions. For this reason, we rely on event studies to study the timing of the effect given that we might expect some lag (see the next section).

13 We calculate these variables from the population data provided by the Surveillance, Epidemiology, and End Results (SEER) Program (Surveillance Epidemiology and End Results Program, 2022).

14 This variable is specific to the dataset (Medicare claims data versus any non-Medicare outcome). For outcomes constructed using the Medicare data, we control for the share of the Medicare beneficiaries that are 65+. For all other outcomes, we control for the share of the state population that is ages 65+ (using the SEER data).

15 These policy dates were collected from the Prescription Drug Abuse Policy System (PDAPS) (Prescription Drug Abuse Policy System, 2022). They reflect the adoption of prescribing practices for acute and emergency care. The PDAPS data end in 2017. We updated the data through 2018.

16 The state policies were collected from the OPTIC database (RAND-USC Schaeffer Opioid Policy Tools and Information Center, 2022a, 2022b) and PDAPS.

17 For estimation of average effects (overall or by time-relative-to-adoption), the estimates are identical to those provided by the imputation estimator discussed in Borusyak et al. (2024). Gardner et al. (2024) conclude that two-stage difference-in-differences and its corresponding inference procedure generally perform well.

18 In addition, including covariates additively in a two-way fixed effects model can also bias the treatment effect estimates, but an imputation approach circumvents these problems as well (Caetano et al., 2022; Powell, 2023).

19 We do not “bin” the endpoints. We estimate normalized relationships for all pre- and post-treatment periods but only report −10 to +20.

20 See Fig. A7 for the same analysis but estimated in levels. This outcome is heavily skewed so we prefer the logged outcome results.

21 There is some evidence of a partial anticipation effect. Anticipation effects would be consistent with a deterrence mechanism since these policies are adopted in advance of implementation, and pill mills would be well-aware of the potential impacts of these policies and the need to change behavior to avoid detection and penalties. Notably, as discussed in Section 4.7, we do not observe other opioid-specific policies adopted in the quarter prior to PMCL adoption.

22 We report $100\times (e^{\hat{\beta }}-1)\%$ (where $\hat{\beta }$ represents the average effect estimate) throughout this paper for logged outcomes.

23 For this latter outcome, the analysis is performed by state*half, not state*quarter.

24 For comparison, Buchmueller and Carey (2018) estimate reductions in the share of beneficiaries receiving 211+ days in a half-year of 4.3 per 1,000 due to the adoption of mandatory-access PDMPs, larger than our estimate of 3.5 / 1,000. See Table 6, Panel B of their paper. While our estimates are smaller, they imply larger proportional reductions since problematic prescribing behavior was less prevalent during our time period.

25 Cerdá et al. (2021) estimate that PMCLs reduce high-dose opioid prescriptions by 5% in the year adoption and increasing to 27% by three years after adoption using pharmacy data.

26 Buchmueller and Carey (2018) estimate that mandatory-access PDMPs decrease the rate of overlapping claims by 5.5 per 1,000. As before though, our estimates imply larger proportional reductions.

27 However, we generally observe impacts immediately for these sub-populations (event studies not shown), suggesting that compositional changes are not driving the results.

28 Mathur and Ruhm (2023) re-analyze and extend the mortality results in the literature, suggesting that the decline in overdoses does not hold as one extends the data or adds control variables. See Abouk et al. (2023) for a discussion about how extending the data through 2018 does not change this relationship.

29 Finding that no policies were enacted in the quarter prior to the PMCLs suggests that the anticipation effects that are observed in some of the event study results are, in fact, anticipation effects and not reflecting concurrent policy-driven movements in opioid prescribing.

30 Our goal is not to replicate the findings in Mallat (2020) and Burton and Churchill (2025). However, one reason that may explain this difference is that our model accounts for the treatment heterogeneity assumption by applying the Gardner (2022) approach, while Mallatt (2020) utilizes a two-way fixed effects model. We have a different data source than the data used in Burton and Churchill (2025).

31 As discussed in Section 3.6, there are other reasons that we may observe larger longer-term reductions than short-term effects since PMCLs may deter opioid dependence which later leads to heavy opioid use and doctor shopping.

32 We drop Arizona. Cerdá et al. (2021) treats Arizona as adopting in April 2018. The findings in this section are not meaningfully affected by the inclusion or exclusion of Arizona.

## Appendix A. Supplementary material

Supplementary data to this article can be found online at https://doi.org/10.1016/j.jpubeco.2025.105515.
