# Supplementary material for: Regulating quasi-legal markets: Evidence from pain management clinic laws
Source: J Public Econ. Author manuscript; Available in PMC 2026 Jan 9. (PMC12782221; doi:10.1016/j.jpubeco.2025.105515)
Supplement: Appendix [file NIHMS2127529-supplement-Appendix.pdf]

Online Appendix

Appendix Figures

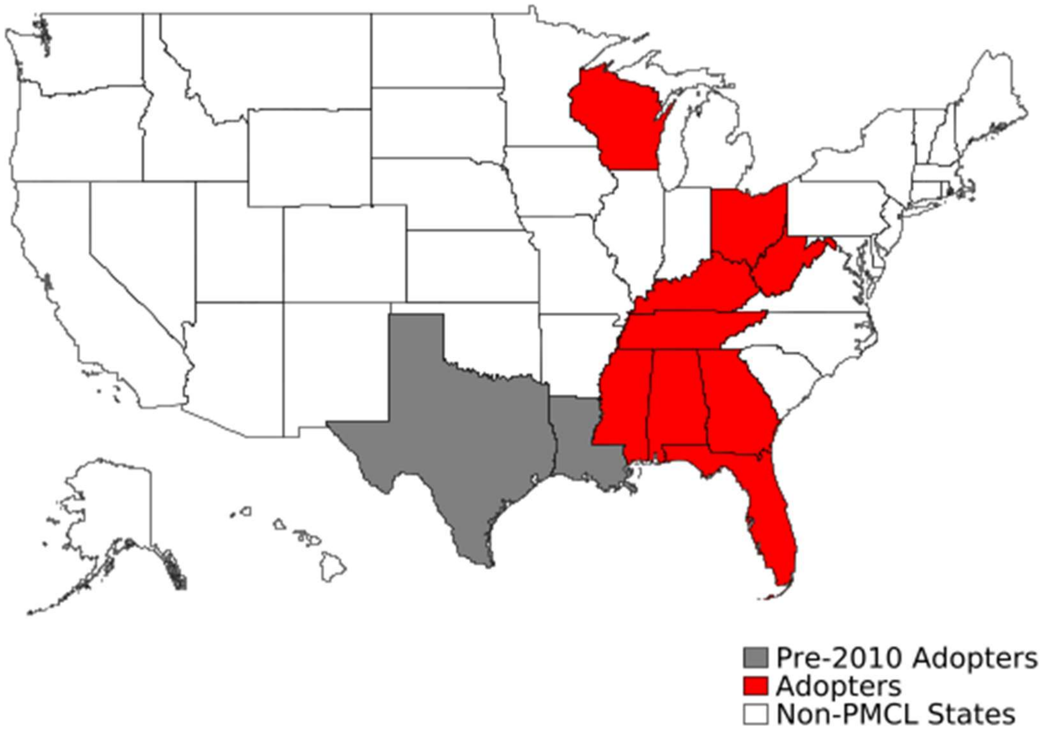

Figure A1: Map of States with PMCLs during Sample Period

Notes: Pre-2010 adopters are excluded from the analysis in this paper. Arizona enacted a PMCL in 2018, but it did not include any inspections or penalties until 2019 so we excluded it from the analysis.

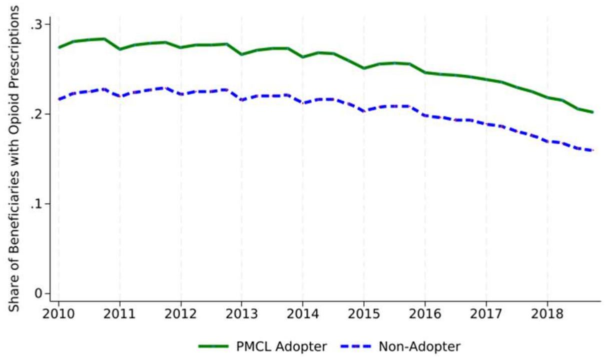

A: Beneficiaries with Opioid Prescriptions

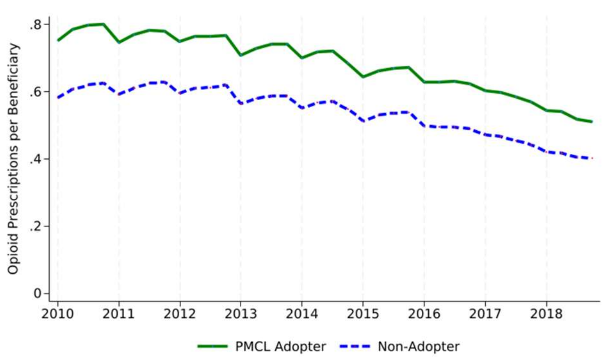

B: Opioid Prescriptions

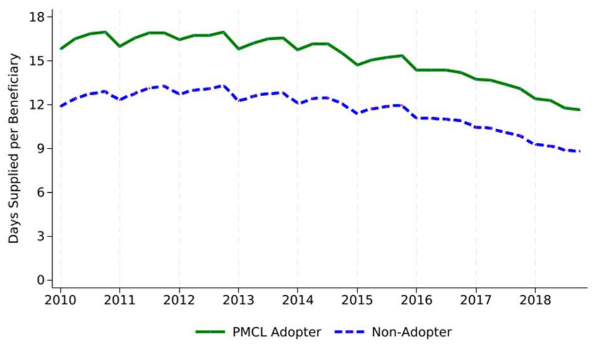

C. Days Supplied

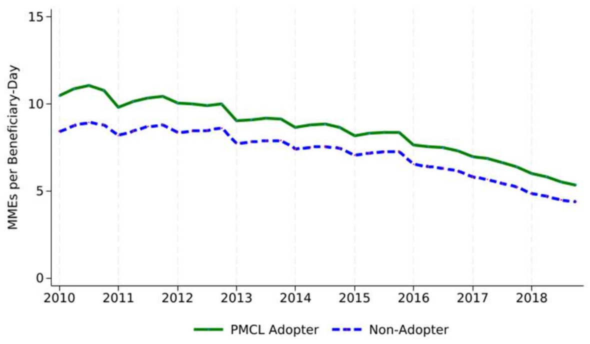

D: Morphine Milligram Equivalents per Day

Figure A2: Time Series Trends for Primary Medicare Prescribing Outcomes

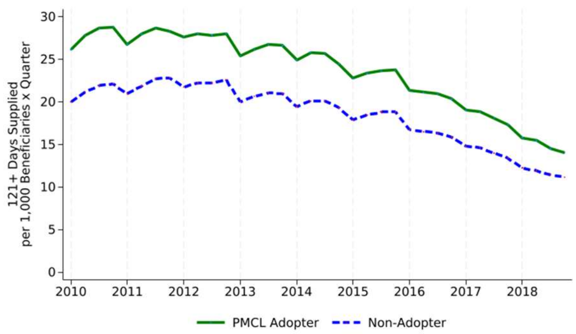

A: 121+ Days per Quarter

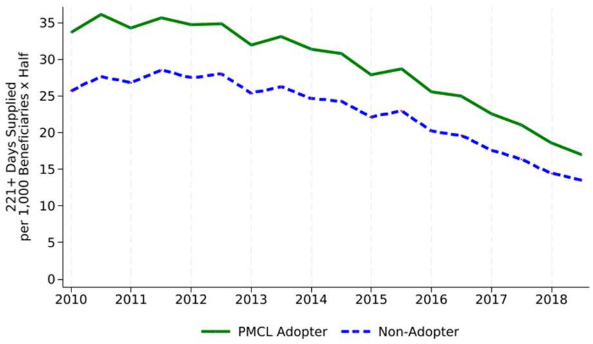

B: 211+ Days Per Half-Year

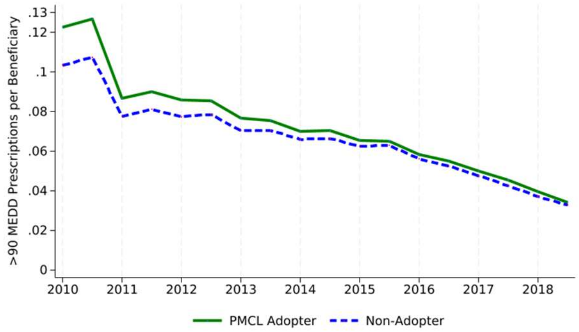

C: > 90 MEDD Prescriptions

Figure A3: Time Series Trends for Heavy Prescribing Outcomes

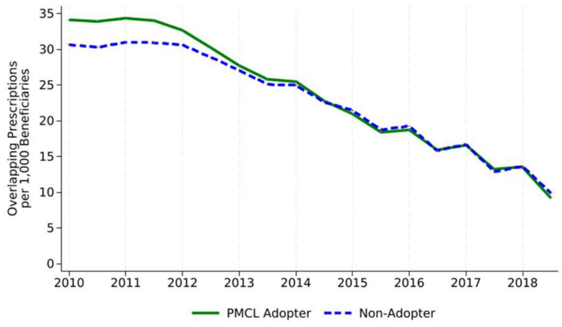

A: Overlapping Claims (per 1,000)

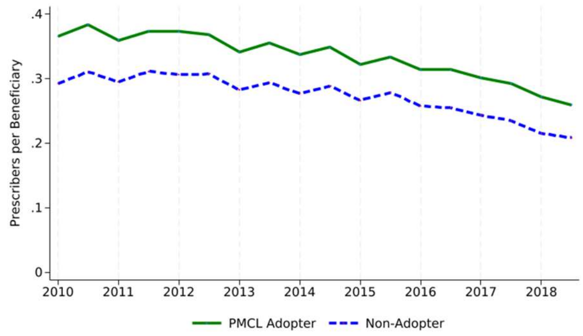

B: Opioid Prescribers

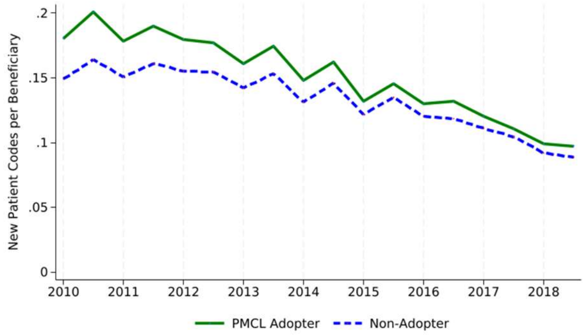

C: New Patient Codes

Figure A4: Time Series Trends for Doctor Shopping Outcomes

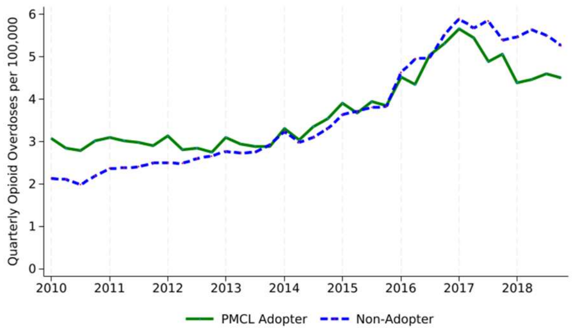

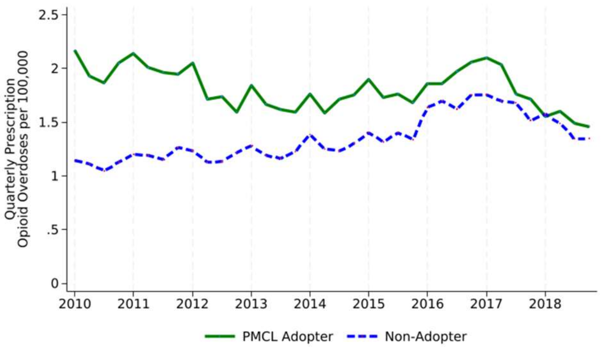

A. Opioids

B. Prescription Opioids

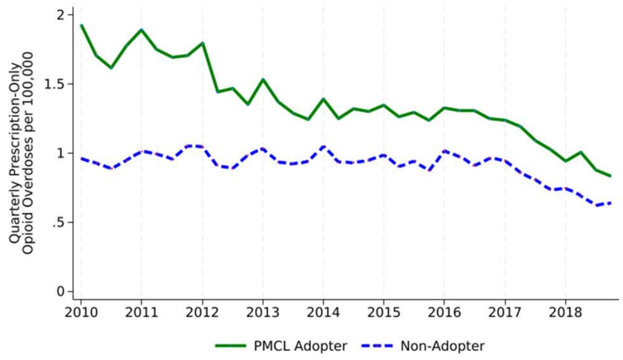

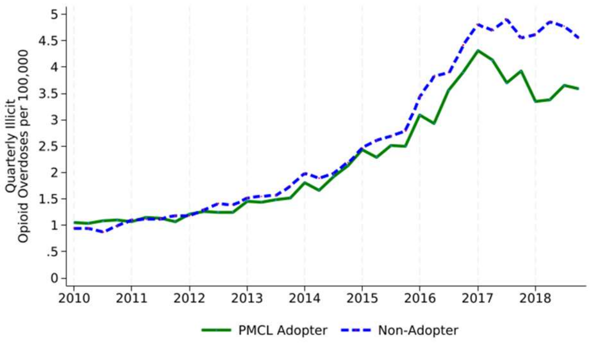

C. Prescription-Only Opioids

D. Illicit Opioids

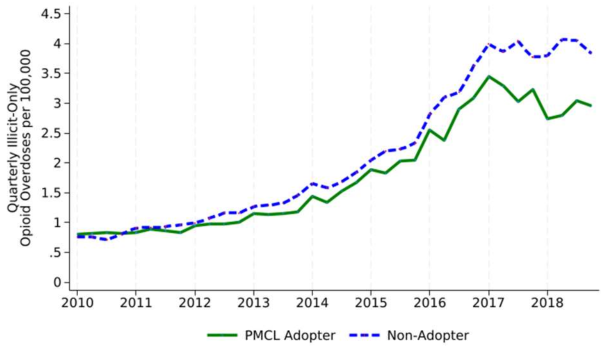

E. Illicit-Only Opioids

Figure A5: Time Series Trends for Overdose Deaths (per 100,000)

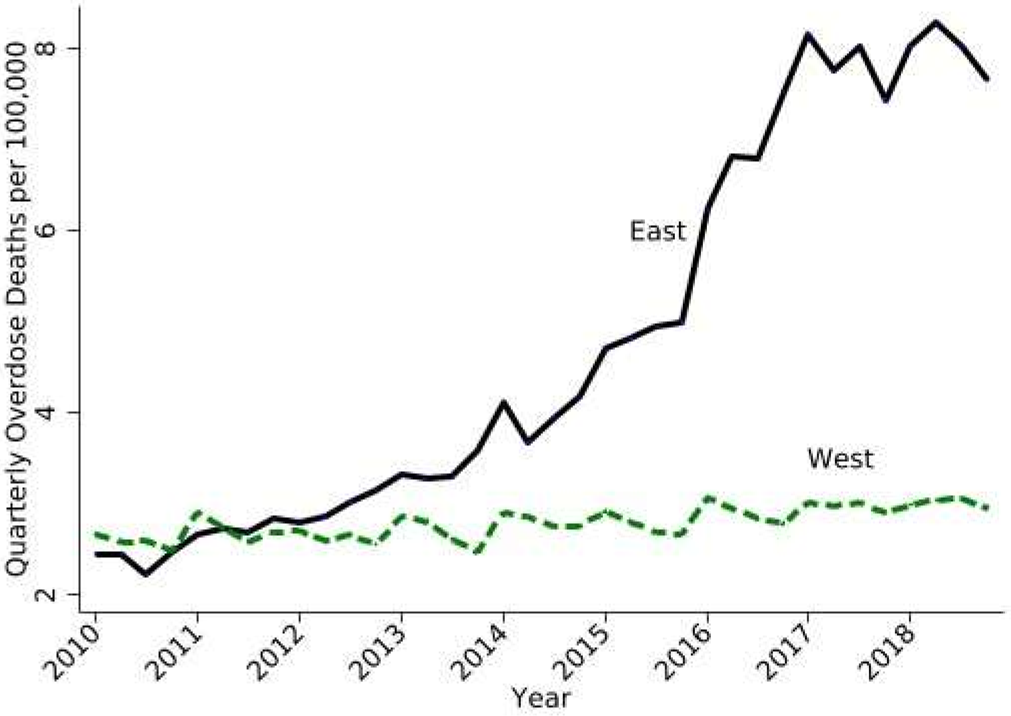

Figure A6: Opioid Overdose Death Trends for Never-Adopters Based on East or West of the Mississippi River by Quarter

Notes: NVSS data 2010-2018. The y-axis represents quarterly opioid-involved overdose deaths per 100,000 among the full population. We exclude Arizona.

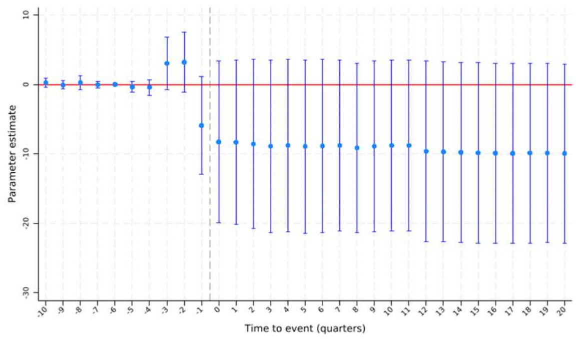

A: Practitioners

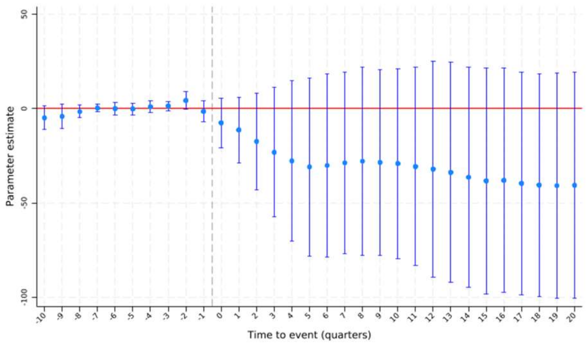

B: Local Pharmacies

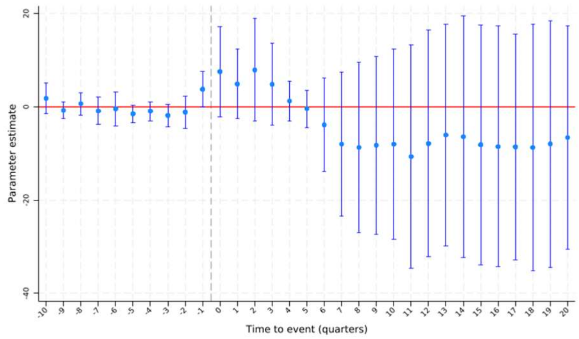

C: Chain Pharmacies

Figure A7: Event Study Estimates For Opioid Distribution by Business Type in Levels (equivalent to Figure 1)

Notes: Estimates and 95% confidence intervals (adjusted for state-level clustering) provided. We use two-stage difference-in-differences, weighted by population size. Outcomes are MMEs per capita distributed to each business type. In the first step, we regress the outcome on state fixed effects, time fixed effects, and covariates using only untreated observations. Only states east of the Mississippi River are included in the analysis. We use the estimates to impute the counterfactuals for the treated units. We regress the difference between the observed outcome and estimated counterfactual on indicators based on quarter-relative-to-adoption (this method does not require normalization). Covariates include share of the state population that is White, share ages 65+, policy variables, and the interaction of the 2004-2009 non-medical OxyContin use rate with year indicators. The policy variables are ACA Medicaid expansion, legal and operational medical marijuana dispensaries, recreational marijuana laws, must-access PDMPs, and opioid prescribing guidelines.

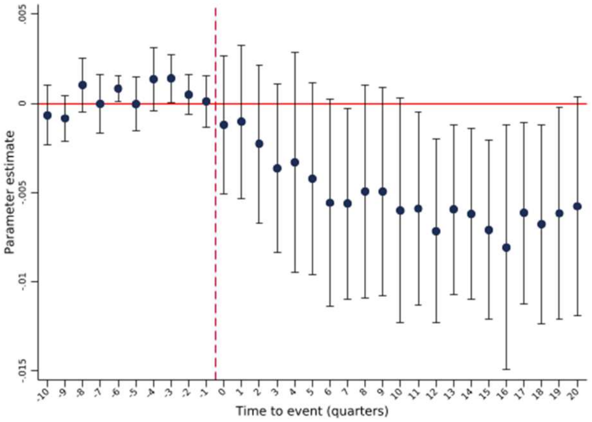

A: Beneficiaries with Opioid Prescriptions

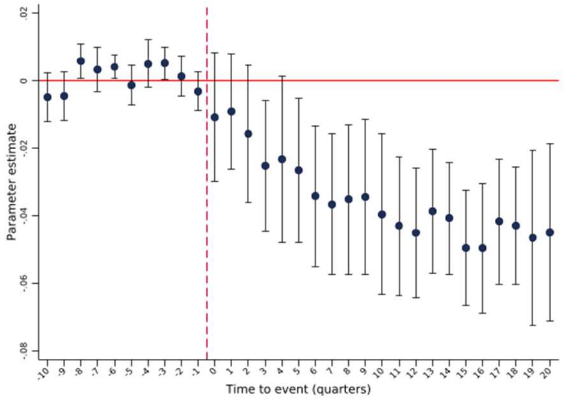

B: Opioid Prescriptions

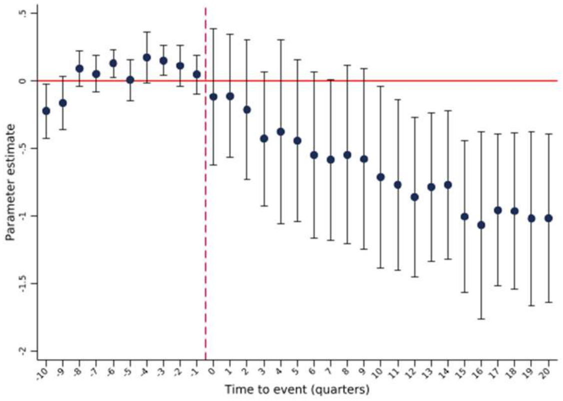

C. Days Supplied

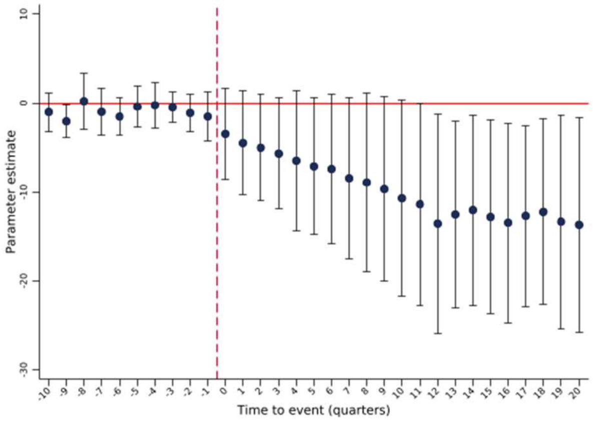

D: Morphine Milligram Equivalents per Day

Figure A8: Event Study Estimates for Per-Beneficiary Opioid Prescription Outcomes

Notes: Estimates and 95% confidence intervals (adjusted for state-level clustering) provided. We use two-stage difference-in-differences, weighted by number of beneficiaries. Outcomes are per Medicare beneficiary. Only states east of the Mississippi River are included in the analysis. In the first step, we regress the outcome on state fixed effects, time fixed effects, and covariates using only untreated observations. We use the estimates to impute the counterfactuals for the treated units. We regress the difference between the observed outcome and estimated counterfactual on indicators based on quarter-relative-to-adoption (this method does not require normalization). Covariates include share of the state population that is White, share of Medicare beneficiaries ages 65+, policy variables, and the interaction of the 2004-2009 non-medical OxyContin use rate with year indicators. The policy variables are ACA Medicaid expansion, legal and operational medical marijuana dispensaries, recreational marijuana laws, must-access PDMPs, and opioid prescribing guidelines. The outcome in Panel A is the share of beneficiaries with at least one opioid prescription in the quarter. The outcome in Panel B is the number of opioid prescriptions divided by the number of beneficiaries. Panels C and D analyze the per-beneficiary total days supplied and MMEs per day, respectively.

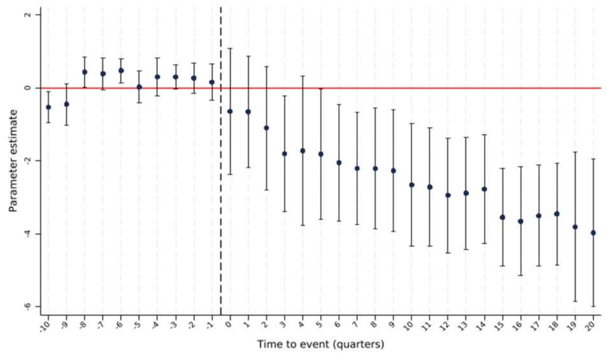

A: 121+ Days per Quarter

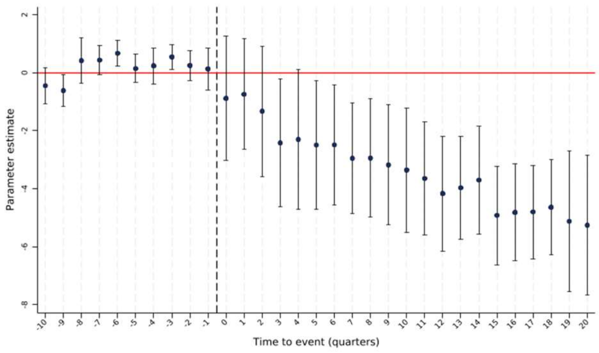

B: 211+ Days Per Half-Year

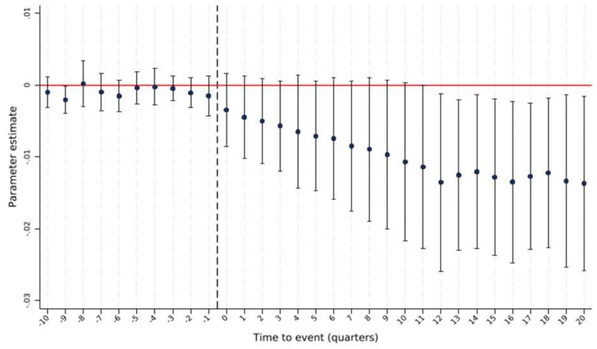

C: > 90 MEDD Prescriptions

Figure A9: Event Study Estimates for Heavy Opioid Use Measures (Per 1,000 Beneficiaries)

Notes: Estimates and 95% confidence intervals (adjusted for state-level clustering) provided. We use two-stage difference-in-differences. Only states east of the Mississippi River are included in the analysis. Outcomes are per 1,000 Medicare beneficiaries. In the first step, we regress the outcome on state fixed effects, time fixed effects, and covariates using only untreated observations. We use the estimates to impute the counterfactuals for the treated units. We regress the difference between the observed outcome and estimated counterfactual on indicators based on quarter-relative-to-adoption (this method does not require normalization). Covariates include share of the state population that is White, share of Medicare beneficiaries ages 65+, policy variables, and the interaction of the 2004-2009 non-medical OxyContin use rate with year indicators. The policy variables are ACA Medicaid expansion, legal and operational medical marijuana dispensaries, recreational marijuana laws, must-access PDMPs, and opioid prescribing guidelines. In Panel B, the analysis is performed using only the 1st and 3rd quarters of each year.

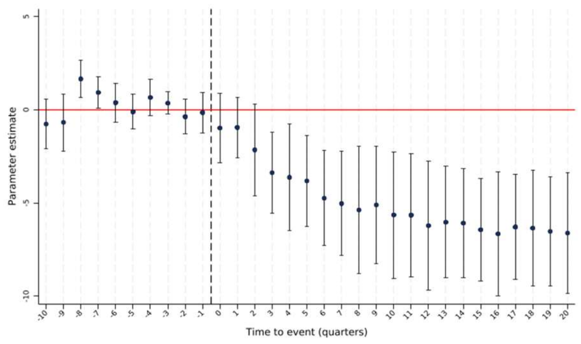

A: Overlapping Claims (per 1,000)

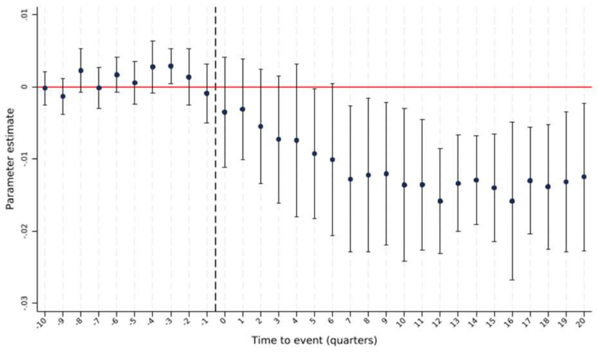

B: Opioid Prescribers

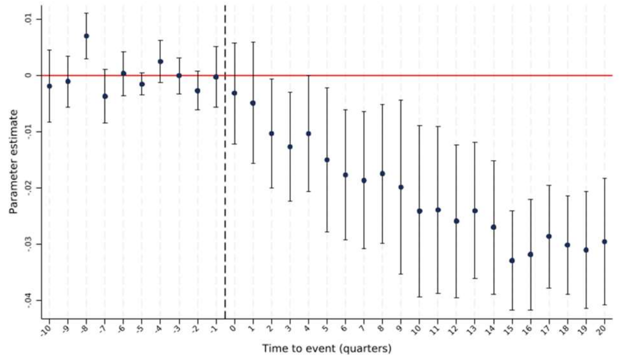

C: New Patient Codes

Figure A10: Event Study Estimates For Doctor Shopping Measures

Notes: Estimates and 95% confidence intervals (adjusted for state-level clustering) provided. We use two-stage difference-in-differences. The outcome in Panel A is per 1,000 Medicare beneficiaries. Outcomes in Panels B and C are per Medicare beneficiary. Only states east of the Mississippi River are included in the analysis. In the first step, we regress the outcome on state fixed effects, time fixed effects, and covariates using only untreated observations. We use the estimates to impute the counterfactuals for the treated units. We regress the difference between the observed outcome and estimated counterfactual on indicators based on quarter-relative-to-adoption (this method does not require normalization). Covariates include share of the state population that is White, share of Medicare beneficiaries ages 65+, policy variables, and the interaction of the 2004-2009 non-medical OxyContin use rate with year indicators. The policy variables are ACA Medicaid expansion, legal and operational medical marijuana dispensaries, recreational marijuana laws, must-access PDMPs, and opioid prescribing guidelines.

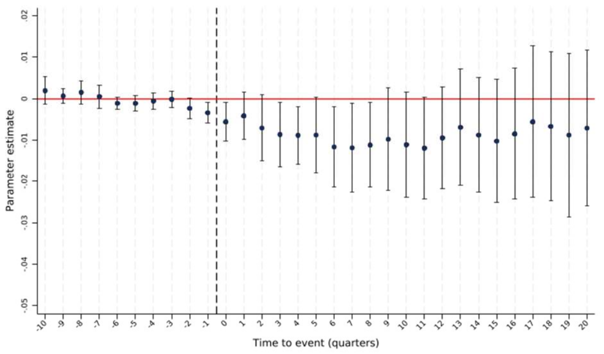

A: 1-7 Day Opioid Prescriptions

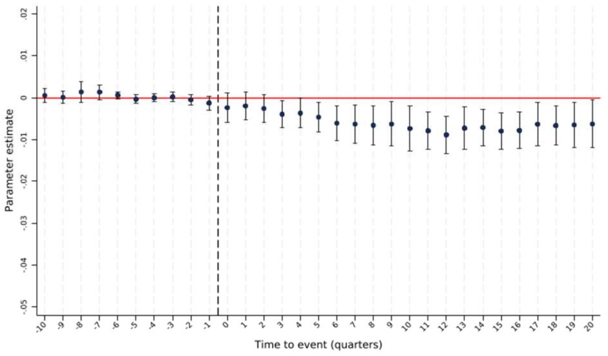

B: 8-14 Day Opioid Prescriptions

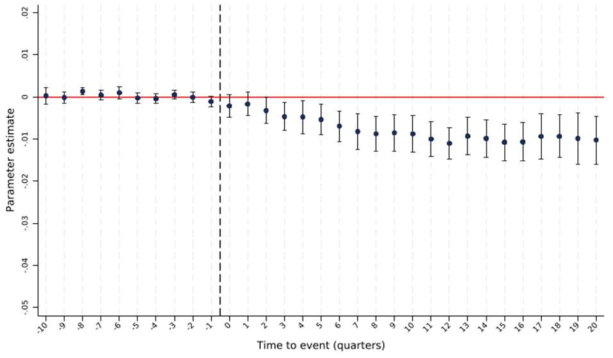

C: 15-21 Day Opioid Prescriptions

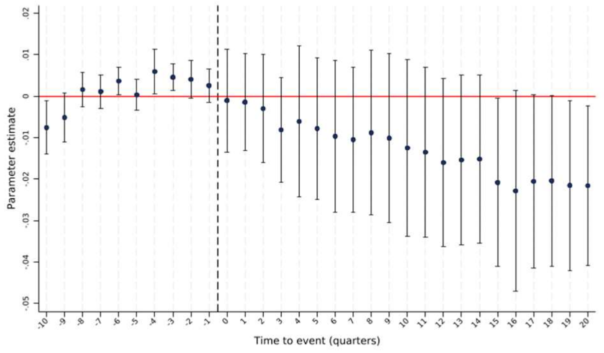

B: 22+ Day Opioid Prescriptions

Figure A11: Event Study Estimates for Opioid Prescriptions by Prescription Length (per beneficiary)

Notes: Estimates and 95% confidence intervals (adjusted for state-level clustering) provided. We use two-stage difference-in-differences. Outcomes are number of prescriptions with specified length per-beneficiary. In the first step, we regress the outcome on state fixed effects, time fixed effects, and covariates using only untreated observations. Only states east of the Mississippi River are included in the analysis. We use the estimates to impute the counterfactuals for the treated units. We regress the difference between the observed outcome and estimated counterfactual on indicators based on quarter-relative-to-adoption (this method does not require normalization). Covariates include share of the state population that is White, share of Medicare beneficiaries ages 65+, policy variables, and the interaction of the 2004-2009 non-medical OxyContin use rate with year indicators. The policy variables are ACA Medicaid expansion, legal and operational medical marijuana dispensaries, recreational marijuana laws, must-access PDMPs, and opioid prescribing guidelines.

QCEW Results

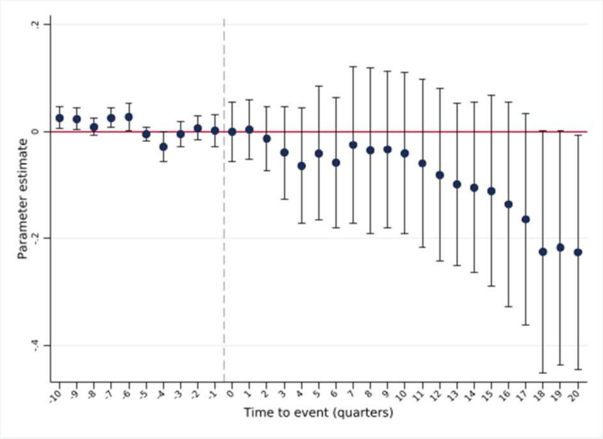

A. All Other Outpatient Clinics

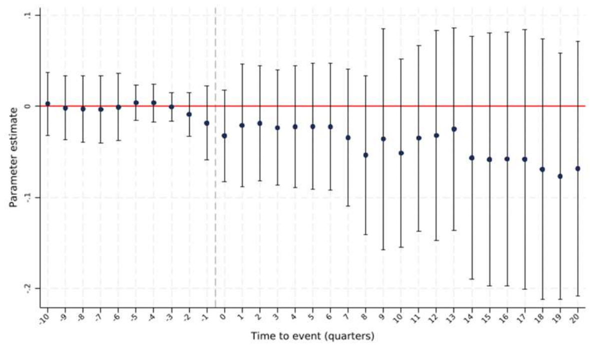

B. Pharmacies

CBP Results

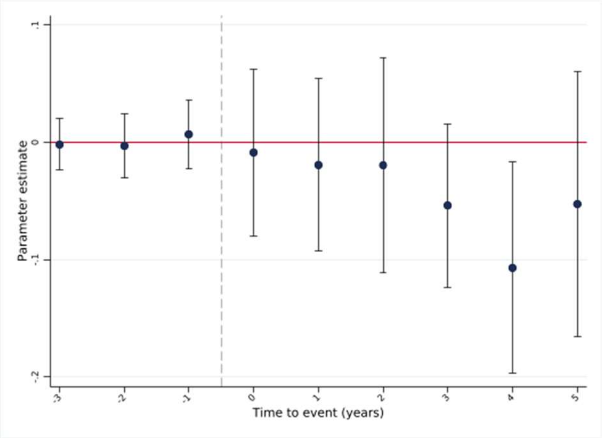

C. All Other Outpatient Clinics

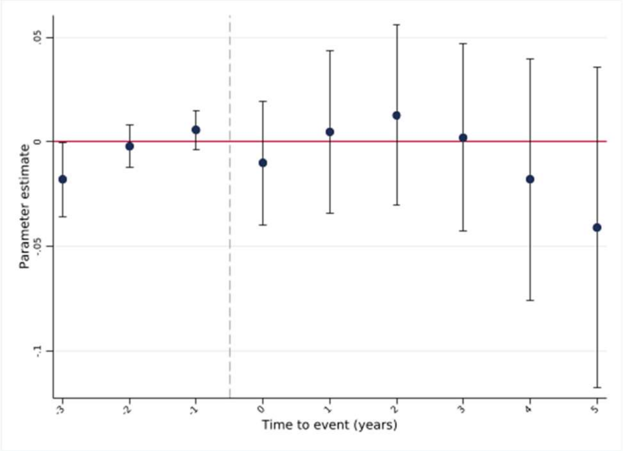

D. Pharmacies

Figure A12: Event Study Estimates For Establishment Counts (per 100,000)

Notes: Estimates and 95% confidence intervals (adjusted for state-level clustering) provided. We use two-stage difference-in-differences, weighted by population sizes. Outcomes are establishment counts per 100,000 population and are logged. In the first step, we regress the outcome on state fixed effects, time fixed effects, and covariates using only untreated observations. Only states east of the Mississippi River are included in the analysis. We use the estimates to impute the counterfactuals for the treated units. We regress the difference between the observed outcome and estimated counterfactual on indicators based on quarter-relative-to-adoption (this method does not require normalization). Covariates include share of the state population that is White, share of Medicare beneficiaries ages 65+, policy variables, and the interaction of the 2004-2009 non-medical OxyContin use rate with year indicators. The policy variables are ACA Medicaid expansion, legal and operational medical marijuana dispensaries, recreational marijuana laws, must-access PDMPs, and opioid prescribing guidelines.

Appendix Tables

Table A1: Detailed Information on PMCL Provisions by State

Category	Description	Alabama	Arizona	Florida	Georgia	Kentucky	Louisiana	Mississippi	Ohio	Tennessee	Texas	West Virginia	Wisconsin	**# of States with this Provision**
	Any PMCL	5/8/2013	4/26/2018	10/1/2010	7/1/2013	7/20/2012	1/1/2006	4/24/2011	5/20/2011	5/30/2011	9/1/2009	6/8/2012	3/19/2016	12
Enforcement and Penalties for Non-compliance	*Any Penalties for Noncompliance*	1/1/2014		10/1/2010	7/1/2013	7/20/2012	5/26/2016	4/24/2011	6/20/2011	5/30/2011	9/1/2010	6/8/2012	11/16/2016	11
	Penalty: Disiplinary Action	1/1/2014		10/1/2010	7/13/2014		5/26/2016	4/24/2011		5/30/2011	9/1/2010	6/10/2016	11/16/2016	9
	Penalty: Criminal Charges	1/1/2014		10/1/2010	7/1/2013	7/20/2012				7/1/2017	9/1/2015			6
	Penalty: Fines	1/1/2014		10/1/2010			5/26/2016		6/20/2011	5/30/2011		6/8/2012	11/16/2016	7
Oversight Over Clinic, Patients, and Physician Practice	*Enables Facility Inpections*			10/1/2010	7/13/2014	7/20/2012	1/1/2008	10/24/2013	3/13/2013	5/30/2011	9/1/2010	6/8/2012		9
	Requirement to Keep Patient Records	1/1/2014		10/1/2010	7/1/2013	7/20/2012	1/1/2006	4/24/2011	6/20/2011	9/30/2011	9/1/2010	6/8/2012		10
	PDMP Registration Required for Pain Clinic Physicians	1/1/2014			7/13/2014	7/20/2012	1/1/2008	4/24/2011	1/1/2015	1/1/2013	9/1/2017	6/10/2016		9
	Requires Physician to Discuss Alternative Forms of Treatment	1/1/2014				7/20/2012	1/1/2006		6/20/2011	5/30/2011	9/1/2010	9/9/2013		7
	Requires Drug Testing Prior to Prescribing				7/1/2013	7/20/2012	1/1/2008		6/20/2011	9/30/2011	9/1/2010	4/1/2015		7
	Requires Physical Examinations Prior to Prescribing	1/1/2014		10/1/2010	7/1/2013	7/20/2012	1/1/2006	4/24/2011	6/20/2011	9/30/2011	9/1/2010	6/8/2012		10
	Explicit Restrictions to Dispensing			10/1/2010	7/1/2013					5/30/2011		6/8/2012	3/19/2016	5
	Medical Director Oversees Operations	1/1/2014		10/1/2010		7/20/2012	1/1/2006	4/24/2011		5/30/2011	9/1/2010	6/8/2012	3/19/2016	9
	Certification/Registration Requirements for Clinic Operation	1/1/2014		10/1/2010	7/1/2013	7/20/2012	1/1/2006	4/24/2011	6/20/2011	5/30/2011	9/1/2010	6/8/2012	3/19/2016	11
Provider Qualifications	*Physician Practice Requirements: Any*	1/1/2014		10/1/2010	7/1/2013	7/20/2012	1/1/2006	4/24/2011	6/20/2011		9/1/2010	6/8/2012		9
	Physician Practice Requirements: No Prior Felony				7/13/2014			10/24/2013	6/20/2011			6/8/2012		4
	Physician Practice Requirements: No Prior License Restrictions				7/1/2013	7/20/2012	1/1/2006		6/20/2011		3/18/2013	6/8/2012		6
	Physician Practice Requirements: No DEA License Denial	1/1/2014		10/1/2010	7/1/2013	7/20/2012	1/1/2006	4/24/2011	6/20/2011		9/1/2010	6/8/2012		9
	Physician Practice Requirements : Certification in Pain Management				7/13/2014			10/24/2013						2
Ownership Qualifications	*Ownership: Any Requirements*	12/29/2013		10/1/2010	7/1/2013	7/20/2012	1/1/2006	4/24/2011	6/20/2011	5/30/2011	9/1/2010	6/8/2012	11/16/2016	11
	Ownership: Physician			10/1/2010	7/1/2013	7/20/2012	1/1/2006	4/24/2011	6/20/2011		9/1/2010	6/8/2012		8
	Ownership: No Prior Felony				7/1/2013		1/1/2006	4/24/2011		5/30/2011	9/1/2010	6/8/2012	11/16/2016	7
	Ownership: No Prior License Restrictions				7/1/2013	7/20/2012	1/1/2006	4/24/2011	6/20/2011		9/1/2010	6/8/2012		7
	Ownership: No DEA License Denial			10/1/2010	7/1/2013	7/20/2012	1/1/2006	4/24/2011	6/20/2011		9/1/2010	6/8/2012		8
	Ownership: Certification in Pain Management				7/1/2013		1/1/2006	10/24/2013	6/20/2011					4

Notes: We follow and confirm PDAPS for most of the dates in this table. We deviate from PDAPS in determining Alabama's ownership requirements and Georgia and Wisconsin's dispensing limitations. Alabama's PMCL has an ownership requirement but is not captured in PDAPS. Alabama's PMCL states that all pain clinic owners in Alabama must be one of the following: (a) a licensed physician in Alabama, (b) a business entity registered with the Alabama Secretary of State's Office, (c) a governmental entity or body, or political subdivision, or any combination thereof, including state universities and schools (Ala. Admin. Code r. 540-X-19-.04). Georgia and Wisconsin's PMCLs have dispensing limitations that are not captured in PDAPS. Georgia's PMCL states that for a pain clinic to dispense controlled substances, the clinic must be registered with the State Board of Pharmacy (Ga Code Ann §43-34-280). Wisconsin's PMCL states that a pain clinic cannot dispense opioids unless it is licensed as a pharmacy or the patient is being treated for a condition or complaint reasonably related to a worker's compensation claim (Wis Stat §50.60 et seq.).

Table A2. Summary of Medicare Prescribing Outcome Variables

Outcome	Description
Any opioid prescribed	Share of beneficiaries receiving at least one opioid prescription in the quarter
Number of opioids prescribed	Number of opioid prescriptions in a quarter per beneficiary
Days Supplied	Number of opioid prescription days supplied in a quarter per beneficiary
MME	Morphine Milligram Equivalent doses prescribed in a quarter per beneficiary-day
121+ days in past 3 months	Number of opioid prescriptions for 121+ days in a quarter per 1,000 beneficiaries
211 + days in past 6 months	Number of opioid prescriptions for 211+ days in a half-year per 1,000 beneficiaries
> 90 MEDD prescriptions	Number of opioid prescriptions with over 90 morphine milligram equivalents per day supplied, per 1,000 beneficiaries
1-7 days	Number of opioid prescriptions for 1-7 days in a quarter per beneficiary
8-14 days	Number of opioid prescriptions for 8-14 days in a quarter per beneficiary
15-21 days	Number of opioid prescriptions for 15-21 days in a quarter per beneficiary
22+ days	Number of opioid prescriptions for 22+ days in a quarter per beneficiary
Number of prescribers	Number of prescribers which patient received opioid prescriptions from in a year per beneficiary
Overlapping prescriptions	Second prescription for the same ingredient more than a week before first prescription should have finished given days supplied in a quarter per beneficiary
New patients	Number of “new patient” codes per quarter. A new patient code has a HCPCS code of 99201, 99202, 99203, 99204, or 99205 in the outpatient or physician (carrier) medical claims

Table A3: Summary Statistics of Variables by Treatment Group Status, First 3 Quarters of 2010

	Adopters	Non-Adopters	P-Value of Difference
**Demographic and Policy Controls**			
Share White	0.739	0.738	0.974
Share 65+	0.721	0.755	0.053
ACA Medicaid Expansion	0.000	0.000	
Medical marijuana dispensaries	0.000	0.000	
Mandatory Access PDMP	0.000	0.000	
Recreational marijuana law	0.000	0.000	
Opioid Prescribing Guidelines	0.111	0.059	0.650
Pre-reformulation OxyContin misuse (%)	0.716	0.685	0.757
**Prescribing Outcomes** (per beneficiary unless otherwise specified)			
Any Opioid Prescription	0.300	0.226	0.001
Opioid Prescriptions	0.853	0.639	0.002
Morphine Milligram Equivalents Per Day	10.984	9.519	0.115
Days Supplied	17.872	12.844	0.002
121+ Days Per Quarter per 1,000	29.037	22.904	0.026
211+ Days per Half-Year per 1,000	36.697	28.500	0.019
Prescriptions >90 MEDD	0.125	0.120	0.700
Overlapping Claims per 1,000	35.548	33.288	0.471
Number of Opioid Prescribers	0.405	0.312	0.003
New Patient Codes	0.208	0.174	0.077
**Mortality Outcomes** (per 100,000)			
Opioids	3.086	2.231	0.037
Prescription Opioids	2.174	1.288	0.014
Illicit Opioids	1.092	0.881	0.249
**Opioid Supply in MME by Establishment Type**			
Practitioners	5.361	1.545	0.112
Local Pharmacies	140.769	82.323	0.001
Chain Pharmacies	129.075	165.977	0.115
**Establishments** (per 100,000)	1.092	0.881	0.249
All Other Outpatient Clinics (QCEW)	1.967	1.627	0.288
Pharmacies (QCEW)	21.586	17.664	0.015
All Other Outpatient Clinics (CBP)	3.342	3.481	0.799
Pharmacies (CBP)	16.630	14.931	0.107

Notes: P-values are not provided for policies in which no states adopted that policy by 2010q3. Summary statistics are provided for 2010q1-2010q3 since the first PMCL in the sample was adopted in 2010q4. “Share 65+” represents the share of the Medicare beneficiaries that are ages 65 and above.

Table A4: Difference-in-Differences Estimates for Medicare Prescribing Outcomes for Sub-Samples

	(1)	(2)	(3)	(4)	(5)	(6)	(7)	(8)	(9)	(10)
	Any Opioids (per beneficiary)	Number of Prescriptions (per beneficiary)	Days Supply (per beneficiary)	MME (per beneficiary)	121+ Days Per Quarter (per 1,000)	211+ Days Per Half-Year (per 1,000)	Prescriptions >90 MEDD (per 1,000)	Overlapping Prescriptions (per 1,000)	Number of Prescribers (per beneficiary)	New Patients (per beneficiary)
**Panel A: Under Age-65**										
PMCL	-0.012***	-0.085***	-2.134***	-3.197*	-7.528***	-9.681***	-32.137**	-14.466***	-0.026***	-0.059***
	(0.004)	(0.023)	(0.731)	(1.736)	(2.377)	(2.784)	(16.214)	(5.103)	(0.008)	(0.015)
Counterfactual Mean	0.438	1.454	34.887	25.344	72.706	88.888	226.725	64.646	0.616	0.356
Implied % Change	-2.824	-5.837	-6.116	-12.614	-10.354	-10.891	-14.174	-22.377	-4.164	-16.567
**Panel B: Ages 65+**										
PMCL	-0.002	-0.011**	-0.117	-0.155	-0.589***	-0.920***	-3.985	-1.615***	-0.004**	-0.008***
	(0.001)	(0.005)	(0.115)	(0.126)	(0.221)	(0.253)	(2.610)	(0.322)	(0.002)	(0.003)
Counterfactual Mean	0.200	0.458	9.760	4.128	10.443	13.332	28.166	12.031	0.247	0.098
Implied % Change	-0.825	-2.480	-1.201	-3.763	-5.636	-6.901	-14.150	-13.421	-1.448	-8.180
**Panel C: Opioid Naïve**										
PMCL	-0.000	-0.002	-0.054*	-0.068*	-0.030**	-0.001	-2.423**	-0.100*	-0.001	-0.002*
	(0.001)	(0.001)	(0.028)	(0.038)	(0.015)	(0.002)	(1.158)	(0.054)	(0.001)	(0.001)
Counterfactual Mean	0.081	0.075	0.850	0.412	0.125	0.010	4.446	0.564	0.063	0.024
Implied % Change	-0.120	-2.486	-6.373	-16.468	-23.861	-12.916	-54.494	-17.714	-1.519	-9.335
**Panel D: Opioid Non-Naïve**										
PMCL	-0.006	-0.054**	-1.322**	-1.747	-5.361***	-6.869***	-14.638	-8.934***	-0.015**	-0.046***
	(0.005)	(0.024)	(0.663)	(1.182)	(2.046)	(2.371)	(11.302)	(2.943)	(0.008)	(0.011)
Counterfactual Mean	0.624	1.864	45.518	26.445	74.945	91.661	206.246	67.749	0.816	0.393
Implied % Change	-0.981	-2.877	-2.905	-6.608	-7.153	-7.494	-7.097	-13.188	-1.804	-11.599

Notes: *10%, **5%, ***1% statistical significance. Standard errors (adjusted for state-level clustering) provided. We use two-stage difference-in-differences, weighted by the number of beneficiaries. Only states east of the Mississippi River are included in the analysis. In the first step, we regress the outcome on state fixed effects, time fixed effects, and covariates using only untreated observations. We use the estimates to impute the counterfactuals for the treated units. We regress the difference between the observed outcome and estimated counterfactual on whether the state had enacted a PMCL. We include a full set of controls: share of the state population that is White, share of Medicare beneficiaries ages 65+, and policy variables. The policy variables are ACA Medicaid expansion, legal and operational medical marijuana dispensaries, recreational marijuana laws, must-access PDMPs, and opioid prescribing guidelines. We also control for the interaction of the 2004-2009 non-medical OxyContin use rate with year indicators. The “Counterfactual Mean” is the mean of the outcome for all treated observations after subtracting off the estimated treatment effect. “Percent Change” is the implied percent change of the estimate given the counterfactual mean.

Table A5: Different Set of Comparison States

	(1) Any Opioids (per beneficiary)	(2) Number of Prescriptions (per beneficiary)	(3) Days Supply (per beneficiary)	(4) MME (per beneficiary)	(5) 121+ Days Per Quarter (per 1,000)	(6) 211+ Days Per Half-Year (per 1,000)	(7) Prescriptions >90 MEDD (per 1,000)	(8) Overlapping Prescriptions (per 1,000)	(9) Number of Prescribers (per beneficiary)	(10) New Patients (per beneficiary)
**Panel A: Main Prescribing Results**										
PMCL	-0.005***	-0.035***	-0.766***	-0.928**	-2.664***	-3.533***	-10.570**	-4.818***	-0.011***	-0.022***
	(0.002)	(0.008)	(0.213)	(0.411)	(0.565)	(0.661)	(4.269)	(1.055)	(0.002)	(0.004)
Counterfactual Mean	0.252	0.676	15.300	8.748	24.228	29.793	70.821	23.651	0.327	0.156
Implied % Change	-2.147	-5.111	-5.004	-10.612	-10.994	-11.859	-14.925	-20.370	-3.267	-14.131
**Panel B: Including Adopters, Indiana, Illinois, and Michigan**										
PMCL	-0.011***	-0.031***	-0.940*	-1.051	-2.046	-3.106	-5.726	-6.976	-0.028***	-0.015**
	(0.003)	(0.006)	(0.511)	(0.778)	(1.572)	(2.291)	(5.453)	(5.269)	(0.010)	(0.006)
Counterfactual Mean	0.258	0.673	15.474	8.871	23.610	29.365	65.976	25.809	0.345	0.149
Implied % Change	-4.441	-4.606	-6.075	-11.853	-8.664	-10.576	-8.679	-27.029	-8.161	-10.351

Notes: *10%, **5%, ***1% statistical significance. Standard errors (adjusted for state-level clustering) provided. See table notes for Tables 3-5 for more information. These results are comparable to Panel C in those tables. We only use adopting states, Indiana, Illinois, and Michigan in Panel B.

Table A6: Replicating Tables 3-5, Excluding Florida

Panel A: Mean Prescribing Outcomes (equivalent to Table 3)				
	(1)	(2)	(3)	(4)
	Any Opioids	Number of Prescriptions	Days Supply	MME
PMCL	-0.005*	-0.037***	-0.509**	-0.357
	(0.002)	(0.010)	(0.241)	(0.241)

Panel B: Heavy Prescribing Outcomes (equivalent to Table 4)			
	(1)	(2)	(3)
	121+ Days Per Quarter (per 1,000)	Days Per Half-Year (per 1,000)	Prescriptions >90 MEDD (per 1,000)
PMCL	-2.428***	-3.244***	-7.357*
	(0.800)	(0.905)	(4.337)

Panel C: Doctor Shopping (equivalent to Table 5)			
	(1)	(2)	(3)
	Overlapping Prescriptions (per 1,000)	Number of Prescribers (per beneficiary)	New Patients (per beneficiary)
PMCL	-4.055***	-0.009***	-0.019***
	(1.176)	(0.003)	(0.005)

Notes: *10%, **5%, ***1% statistical significance. Standard errors (adjusted for state-level clustering) provided. See table notes for Tables 3-5 for more information. These results are comparable to Panel C in those tables. Florida excluded from analyses.

Table A7: Concurrent Policy Adoption Test

	(1) Medicaid Expansion	(2) Mandatory Access PDMP	(3) Opioid Prescribing Guidelines	(4) Naloxone Access Law	(5) E-Prescribing Mandate
PMCL	-0.344*	0.006	0.328**	0.081	-0.101
	(0.199)	(0.238)	(0.152)	(0.111)	(0.079)

	(6) Good Samaritan	(7) Prescribing Limits	(8) Medical Cannabis Law	(9) Recreational Cannabis Law
PMCL	-0.006	-0.263***	-0.196**	-0.013
	(0.098)	(0.068)	(0.083)	(0.019)

Notes: *10%, **5%, ***1% statistical significance. Standard errors (adjusted for state-level clustering) provided. We use two-stage difference-in-differences, weighted by population. In the first step, we regress the outcome on state fixed effects and time fixed effects. Only states east of the Mississippi River are included in the analysis. We use the estimates to impute the counterfactuals for the treated units. We regress the difference between the observed outcome and estimated counterfactual on whether the state had enacted a PMCL. Covariates include share of the state population that is White and share of Medicare beneficiaries ages 65+. We do not condition on other policy variables in this analysis (results are similar if we include the pre-reformulation rate of OxyContin misuse interacted with year indicators). Medical cannabis law means the presence of legal and operational dispensaries in the state.

Table A8: Difference-in-Differences Estimates for Establishment Counts (per 100,000)

	(1)	(2)	(3)	(4)
	QCEW	QCEW	CBP	CBP
	All Other Outpatient Clinics	Pharmacies	All Other Outpatient Clinics	Pharmacies
**Panel A: State and Time Fixed Effects**				
PMCL	-0.129*	-0.095***	-0.041	-0.003
	(0.073)	(0.036)	(0.035)	(0.035)
**Panel B: + Demographic and Policy Controls**				
PMCL	-0.142*	-0.062	-0.032	0.000
	(0.079)	(0.040)	(0.037)	(0.030)
**Panel C: + OxyContin Misuse**				
PMCL	-0.148*	-0.054	-0.032	-0.001
	(0.080)	(0.043)	(0.038)	(0.029)

Notes: *10%, **5%, ***1% statistical significance. Standard errors (adjusted for state-level clustering) provided. We use two-stage difference-in-differences, weighted by total population. Outcomes are establishment counts per 100,000 population and are logged. Only states east of the Mississippi River are included in the analysis. In the first step, we regress the outcome on state fixed effects, time fixed effects, and covariates using only untreated observations. We use the estimates to impute the counterfactuals for the treated units. We regress the difference between the observed outcome and estimated counterfactual on whether the state had enacted a PMCL. "Demographic and Policy Controls" include share of the state population that is White, share of Medicare beneficiaries ages 65+, and policy variables. The policy variables are ACA Medicaid expansion, legal and operational medical marijuana dispensaries, recreational marijuana laws, must-access PDMPs, and opioid prescribing guidelines. Panel C includes controls for the interaction of the 2004-2009 non-medical OxyContin use rate with year indicators. The "Counterfactual Mean" is the mean of the outcome for all treated observations after subtracting off the estimated treatment effect. "Percent Change" is the implied percent change of the estimate given the counterfactual mean. QCEW=Quarterly Census of Employment and Wages; CBP=County Business Patterns.

Table A9: Difference-in-Differences Estimates for Mortality Results (without Drake and Ruhm 2023 correction)

	(1) Opioids	(2) Prescription Opioids	(3) Prescription Opioids Only	(4) Illicit Opioids	(5) Illicit Opioids Only
**Panel A: State and Time Fixed Effects**					
PMCL	-0.680	-0.477***	-0.439***	-0.311	-0.273
	(0.484)	(0.174)	(0.168)	(0.430)	(0.393)
Counterfactual Mean	4.103	2.053	1.594	2.475	2.016
Implied % Change	-16.567	-23.219	-27.518	-12.554	-13.525
**Panel B: + Demographic and Policy Controls**					
PMCL	-0.694	-0.412*	-0.386**	-0.277	-0.251
	(0.706)	(0.218)	(0.158)	(0.621)	(0.545)
Counterfactual Mean	4.117	1.989	1.541	2.442	1.995
Implied % Change	-16.852	-20.732	-25.050	-11.361	-12.596
**Panel C: + OxyContin Misuse**					
PMCL	-0.704	-0.416*	-0.387**	-0.283	-0.254
	(0.709)	(0.223)	(0.159)	(0.620)	(0.542)
Counterfactual Mean	4.128	1.992	1.542	2.448	1.997
Implied % Change	-17.063	-20.885	-25.078	-11.568	-12.704

Notes: *10%, **5%, ***1% statistical significance. Standard errors (adjusted for state-level clustering) provided. Overdose rates are not adjusted using Drake and Ruhm (2023) but are otherwise comparable to Table 7. We use two-stage difference-in-differences, weighted by population size. Only states east of the Mississippi River are included in the analysis. In the first step, we regress the outcome on state fixed effects, time fixed effects, and covariates using only untreated observations. We use the estimates to impute the counterfactuals for the treated units. We regress the difference between the observed outcome and estimated counterfactual on whether the state had enacted a PMCL. “Demographic and Policy Controls” include share of the state population that is White, share of Medicare beneficiaries ages 65+, and policy variables. The policy variables are ACA Medicaid expansion, legal and operational medical marijuana dispensaries, recreational marijuana laws, must-access PDMPs, and opioid prescribing guidelines. Panel C includes controls for the interaction of the 2004-2009 non-medical OxyContin use rate with year indicators. The “Counterfactual Mean” is the mean of the outcome for all treated observations after subtracting off the estimated treatment effect. “Percent Change” is the implied percent change of the estimate given the counterfactual mean.

Table A10: Difference-in-Differences Estimates for Mortality Results by Substance Involved

	(1)<br>Natural/Semisynthetic	(2)<br>Methadone	(3)<br>Heroin	(4)<br>Synthetic
PMCL	-0.403**	-0.153***	-0.225	-0.481
	(0.205)	(0.051)	(0.224)	(0.610)
Counterfactual Mean	1.975	0.467	1.334	2.378
Implied % Change	-20.420	-32.710	-16.870	-20.225

Notes: *10%, **5%, ***1% statistical significance. Standard errors (adjusted for state-level clustering) provided. We use two-stage difference-in-differences, weighted by population size. Only states east of the Mississippi River are included in the analysis. In the first step, we regress the outcome on state fixed effects, time fixed effects, and covariates using only untreated observations. We use the estimates to impute the counterfactuals for the treated units. We regress the difference between the observed outcome and estimated counterfactual on whether the state had enacted a PMCL. We include the following controls: share of the state population that is White, share of population ages 65+, and policy variables. The policy variables are ACA Medicaid expansion, legal and operational medical marijuana dispensaries, recreational marijuana laws, must-access PDMPs, and opioid prescribing guidelines. We also control for the interaction of the 2004-2009 non-medical OxyContin use rate with year indicators. The "Counterfactual Mean" is the mean of the outcome for all treated observations after subtracting off the estimated treatment effect. "Percent Change" is the implied percent change of the estimate given the counterfactual mean.

Table A11: Difference-in-Differences Estimates for Mortality Results by Age

	(1) Opioids	(2) Prescription Opioids	(3) Prescription Opioids Only	(4) Illicit Opioids	(5) Illicit Opioids Only
**Panel A: Ages 18-64**					
PMCL	-1.356	-0.747**	-0.675***	-0.629	-0.555
	(1.197)	(0.376)	(0.259)	(1.020)	(0.884)
Counterfactual Mean	7.628	3.532	2.667	4.730	3.862
Implied % Change	-17.784	-21.150	-25.311	-13.295	-14.368
**Panel B: Ages 65+**					
PMCL	-0.111	-0.030	-0.014	-0.079	-0.063
	(0.086)	(0.050)	(0.032)	(0.066)	(0.049)
Counterfactual Mean	0.908	0.545	0.445	0.401	0.301
Implied % Change	-12.240	-5.425	-3.229	-19.602	-21.022

Notes: *10%, **5%, ***1% statistical significance. Standard errors (adjusted for state-level clustering) provided. Results are comparable to those in Table 7. We use two-stage difference-in-differences, weighted by population size. Only states east of the Mississippi River are included in the analysis. In the first step, we regress the outcome on state fixed effects, time fixed effects, and covariates using only untreated observations. We use the estimates to impute the counterfactuals for the treated units. We regress the difference between the observed outcome and estimated counterfactual on whether the state had enacted a PMCL. We include the following controls: share of the state population that is White, share of population ages 65+, and policy variables. The policy variables are ACA Medicaid expansion, legal and operational medical marijuana dispensaries, recreational marijuana laws, must-access PDMPs, and opioid prescribing guidelines. We also control for the interaction of the 2004-2009 non-medical OxyContin use rate with year indicators. The "Counterfactual Mean" is the mean of the outcome for all treated observations after subtracting off the estimated treatment effect. "Percent Change" is the implied percent change of the estimate given the counterfactual mean.

Table A12: Comparison with Cerdá et al. (2021)

	Panel A: Main Mortality Results				
	(1)	(2)	(3)	(4)	(5)
	Opioids	Prescription Opioids	Prescription Opioids Only	Illicit Opioids	Illicit Opioids Only
PMCL	-0.949	-0.495**	-0.435***	-0.478	-0.416
	(0.769)	(0.237)	(0.162)	(0.658)	(0.570)
Counterfactual Mean	4.952	2.302	1.739	3.059	2.495
Implied % Change	-19.173	-21.521	-25.010	-15.629	-16.692

	Panel B: Including West as Comparison States				
PMCL	-0.113	-0.265	-0.330**	0.231	0.166
	(0.490)	(0.166)	(0.141)	(0.419)	(0.379)
Counterfactual Mean	4.116	2.072	1.635	2.350	1.912
Implied % Change	-2.755	-12.805	-20.213	9.814	8.703

Notes: *10%, **5%, ***1% statistical significance. Standard errors (adjusted for state-level clustering) provided. Panel A results are identical to those in Table 7 Panel C. Only states east of the Mississippi River are included in the analysis. Panel B results include states west of the Mississippi River (except for Arizona) as part of the comparison group. We use two-stage difference-in-differences, weighted by population size. In the first step, we regress the outcome on state fixed effects, time fixed effects, and covariates using only untreated observations. We use the estimates to impute the counterfactuals for the treated units. We regress the difference between the observed outcome and estimated counterfactual on whether the state had enacted a PMCL. We include the following controls: share of the state population that is White, share of population ages 65+, and policy variables. The policy variables are ACA Medicaid expansion, legal and operational medical marijuana dispensaries, recreational marijuana laws, must-access PDMPs, and opioid prescribing guidelines. We also control for the interaction of the 2004-2009 non-medical OxyContin use rate with year indicators. The "Counterfactual Mean" is the mean of the outcome for all treated observations after subtracting off the estimated treatment effect. "Percent Change" is the implied percent change of the estimate given the counterfactual mean.

Table A13: Importance of Including/Excluding West for Prescribing Outcomes

	(1) Any Opioids (per beneficiary)	(2) Number of Prescriptions (per beneficiary)	(3) Days Supply (per beneficiary)	(4) MME (per beneficiary)	(5) 121+ Days Per Quarter (per 1,000)	(6) 211+ Days Per Half-Year (per 1,000)	(7) Prescriptions >90 MEDD (per 1,000)	(8) Overlapping Prescriptions (per 1,000)	(9) Number of Prescribers (per beneficiary)	(10) New Patients (per beneficiary)
**Panel A: Main Prescribing Results**										
PMCL	-0.005***	-0.035***	-0.766***	-0.928**	-2.664***	-3.533***	-10.570**	-4.818***	-0.011***	-0.022***
	(0.002)	(0.008)	(0.213)	(0.411)	(0.565)	(0.661)	(4.269)	(1.055)	(0.002)	(0.004)
Counterfactual Mean	0.252	0.676	15.300	8.748	24.228	29.793	70.821	23.651	0.327	0.156
Implied % Change	-2.147	-5.111	-5.004	-10.612	-10.994	-11.859	-14.925	-20.370	-3.267	-14.131
**Panel B: Including West as Comparison States**										
PMCL	-0.006***	-0.028***	-0.746***	-0.814**	-2.189***	-2.877***	-9.479***	-3.867***	-0.011***	-0.018***
	(0.002)	(0.011)	(0.166)	(0.325)	(0.613)	(0.747)	(3.342)	(0.905)	(0.002)	(0.003)
Counterfactual Mean	0.253	0.670	15.280	8.633	23.754	29.137	69.729	22.700	0.328	0.152
Implied % Change	-2.468	-4.236	-4.880	-9.429	-9.215	-9.876	-13.594	-17.036	-3.373	-12.127

Notes: *10%, **5%, ***1% statistical significance. Standard errors (adjusted for state-level clustering) provided. We use two-stage difference-in-differences, weighted by the number of beneficiaries. Only states east of the Mississippi River are included in the Panel A analysis (identical to the main results in the paper). In the Panel B analysis, states in the west are also included (except Arizona). In the first step, we regress the outcome on state fixed effects, time fixed effects, and covariates using only untreated observations. We use the estimates to impute the counterfactuals for the treated units. We regress the difference between the observed outcome and estimated counterfactual on whether the state had enacted a PMCL. Covariates include share White, share of Medicare beneficiaries ages 65+, and policy variables. The policy variables are ACA Medicaid expansion, legal and operational medical marijuana dispensaries, recreational marijuana laws, must-access PDMPs, and opioid prescribing guidelines. We also include controls for the interaction of the 2004-2009 non-medical OxyContin use rate with year indicators. The "Counterfactual Mean" is the mean of the outcome for all treated observations after subtracting off the estimated treatment effect. "Percent Change" is the implied percent change of the estimate given the counterfactual mean.
